# Modulatory effects of CeO_2_ nanoparticles on bleomycin-induced active pulmonary disease processes in animal and human airway epithelium models

**DOI:** 10.1186/s12989-026-00658-9

**Published:** 2026-01-16

**Authors:** Chang Guo, Alison Buckley, Sarah Robertson, Adam Laycock, Xianjin Cui, Eugenia Valsami-Jones, Tim Gant, Martin O. Leonard, Rachel Smith

**Affiliations:** 1 Toxicology Department, Radiation, Chemicals, Climate and Environmental Hazards Directorate (RCE), UK Health Security Agency (UKHSA), Harwell Campus, Oxfordshire, OX11 0RQ UK; 2 The National Institute for Health Research Health Protection Research Unit (NIHR HPRU) in Environmental Exposures and Health (EEH) at Imperial College London in Partnership with UKHSA, Oxfordshire, UK; 3 Public Health Scotland (PHS), Gyle Square, 1 South Gyle Crescent, Edinburgh, EH12 9EB UK; 4 School of Geography, Earth and Environmental Sciences, University of Birmingham, Birmingham, B15 2TT UK

**Keywords:** Cerium, Inhalation, Bleomycin, Pulmonary disease process, Air-liquid interface (ALI)

## Abstract

**Background:**

Understanding the impacts of inhaled insoluble nanomaterials as they are encountered in the environment and workplace, in injured lungs remains limited, particularly with respect to their role in the progression or mitigation of lung pathology. While some studies suggest potential protective effects of cerium(IV) oxide nanoparticles (CeO_2_NPs) under certain conditions, their influence during active disease processes is unclear. This study builds on prior work to investigate the effects of CeO_2_NP aerosols on bleomycin-induced pulmonary injury and active disease processes.

**Method:**

To establish conditions of active pulmonary disease processes, bleomycin was used in both animal and airway epithelium models. Male Sprague-Dawley rats were intratracheally instilled with bleomycin or saline (control) followed by nose-only inhalation exposure to CeO_2_NP aerosols (diameter of ~ 43 nm) or control for 3 h per day for 4 days per week for one or two weeks. At three days postexposure, the animals were sacrificed for analysis of bronchoalveolar lavage (BAL) fluid, lung histopathology and global mRNA expression. Comparative in vitro studies were conducted to investigate biological responses at the cellular level, using 3D human small airway epithelium cultures (SmallAir™) exposed to CeO_2_NP aerosols (with a diameter of ~ 86 nm) at the air-liquid-interface at deposition doses comparable to those received in vivo in the small airway.

**Results:**

In vivo, bleomycin treatment resulted in an increase in total BAL cells and fibrotic staining, and significant induction of inflammatory and oxidative stress, as shown by mRNA sequencing analysis. One week of exposure to CeO_2_NPs modified these responses by attenuating fibrotic staining and reducing the expression of genes associated with lung function, inflammation and epithelial-mesenchymal transition (EMT). In vitro, CeO_2_NP exposure modulated some bleomycin-induced cellular responses, although these models do not fully capture the complexity of whole body and tissue systems, highlighting limitations and considerations for future in vitro exposure studies.

**Conclusions:**

In this study, inhaled CeO_2_NPs modulated lung injury responses in the context of active disease, with both potential protective effects and adverse outcomes. These findings demonstrate that the timing of CeO_2_NP exposure relative to disease progression is critical and highlight the need for hazard assessment frameworks to consider context-dependent effects, particularly in the presence of pre-existing lung injury.

**Supplementary Information:**

The online version contains supplementary material available at 10.1186/s12989-026-00658-9.

## Background

Nanomaterials (NMs) exhibit distinctive mechanical, chemical, electrical, and optical properties, facilitating their application across a broad range of fields. However, concerns about their potential impact on public health remain, posing challenges to the development of effective hazard and risk assessment strategies. Among NMs, cerium(IV) oxide nanoparticles (CeO_2_NPs) have received considerable attention in nanotechnology and nanomedicine because of their exceptional catalytic properties, which facilitate both oxidation and reduction reactions (i.e., redox reactions). These properties make CeO_2_NPs highly valuable as catalysts in various applications, including for coating oxygen-sensitive materials, enhancing fuel cell performance, and reducing harmful components of diesel exhaust emissions [1].

Despite these benefits, CeO_2_NPs raise significant environmental and health concerns. Their use as diesel additives has been linked to increased particulate matter (PM) emissions and the formation of self-nucleated metallic particles, contributing to their detection in ambient air, particularly near roadways [2, 3]. Such incidental exposure has raised public health concerns, as inhalation biodistribution studies indicate that CeO_2_NPs accumulate in pulmonary tissue, exhibit slow clearance, and cause persistent inflammation and other pulmonary effects [4–6].

Numerous in vivo studies using pulmonary instillation or inhalation approaches have demonstrated that CeO_2_NPs induce toxic pulmonary effects, including alveolar epithelial hyperplasia, lung inflammation, damage to the air-blood barrier, and fibrosis [7–12]. However, most studies have used animal models representing healthy individuals, overlooking the reality that billions of people globally live with chronic health conditions such as diabetes, chronic liver disease, asthma, chronic obstructive pulmonary disease (COPD), and cardiovascular disorders. Moreover, a significant number of individuals may suffer from undiagnosed subclinical conditions. Accounting for these diverse health profiles is crucial, as NM-induced toxicity can be amplified in pathophysiological models (those with preexisting diseases) compared with healthy models [13].

Research investigating CeO_2_NP toxicity in individuals with preexisting medical conditions has been less extensive than that investigating other NMs, such as SiO_2_, TiO_2_, ZnO, and carbon-based NMs. However, a few studies suggest important interactions in disease models. For example, in a murine model of asthma induced by house dust mites (HDMs), CeO_2_NPs delivered intranasally shifted the pulmonary response toward a type II inflammatory environment [14, 15]. In a separate study using a 5xFAD transgenic mouse model of Alzheimer’s disease, subacute inhalation exposure to CeO_2_NPs had no significant effect on neuroinflammation or oxidative stress, highlighting the need for further context-specific investigations [16].

In addition to having environmental and public health implications, CeO_2_NPs hold immense promise in biomedicine because of their antioxidant properties. These properties suggest potential applications in treating a variety of oxidative stress-related conditions [17], including neurological disorders [18], ophthalmic diseases [19], diabetes [20], and cardiovascular diseases [21]. Furthermore, CeO_2_NPs, with their high surface area and modifiability, have been used as delivery vehicles for therapeutic agents. For example, CeO_2_NPs were utilised for the delivery of microRNA-146a, which has demonstrated protective effects against severe burn-induced remote acute lung injury by modulating the anti-inflammatory TLR4/NF-κB signalling pathway [22]. This approach has been tested in models of bleomycin-induced fibrotic lung injury and lipopolysaccharide (LPS)-induced sterile inflammatory lung injury [23–26].

A comprehensive understanding of the mechanisms underlying both the beneficial and toxic effects of CeO_2_NPs is essential to ensure their safe and effective use. Previously, we assessed the pulmonary toxicity of inhaled nanosized cerium oxide aerosols in Sprague Dawley rats and highlighted the critical influence of aerosol particle size on toxicity outcomes [11]. Additionally, in a separate study, we investigated the molecular mechanisms of CeO_2_NPs by incubating alveolar type II epithelial cells (A549) under oxidative stress conditions, revealing that the TGF-β signalling pathway plays a key role in their protective effects [27]. Building on our previous in vivo research on CeO_2_NP inhalation, this study extends this investigation to utilize both in vivo and in vitro models of bleomycin-induced pulmonary injury and active disease processes. The aims of this study were to elucidate how CeO_2_NPs interact within complex disease environments and improve our understanding of the potential health risks associated with inhalation.

## Methods

### Cerium(IV) oxide nanoparticles (CeO_2_NPs)

For both in vivo and in vitro exposure, CeO_2_NPs were synthesized and purified following previously described methods [28], with further details provided in the Supplementary Information. For primary particle sizing, the CeO_2_NP stock suspensions were drop-cast onto transmission electron microscope (TEM) grids (carbon film on 400 mesh copper grids, AGS160-4, Agar Scientific Ltd, Stansted, Essex, UK) for high resolution TEM imaging (JEOL 3000 F, JEOL Inc. Tokyo, Japan) and subsequent sizing via the image processing software ImageJ [29]. Further details of the sizing method and representative images are given in the Supplementary Information. The agglomeration state of the primary particles in the stock suspensions was investigated via dynamic light scattering (DLS, Malvern Zetasizer Nano, Malvern, UK) and confirmed via TEM image analysis. The particle sizes are summarised in Table 1.

### In vivo exposure and endpoint analysis

#### Bleomycin injury animal model

The experiments were performed within the legal framework of the United Kingdom under a project licence granted by the Home Office of His Majesty’s Government. All procedures involving the animals were performed in accordance with the Animals (Scientific Procedures) Act 1986 and were approved by the local AWERB committee. Male Sprague-Dawley (SD) rats (10–12 weeks, 250–320 g) were purchased from Harlan, UK.

Bleomycin sulphate from Streptomyces verticillus (MERCK, # B5507) was dissolved in sterile phosphate buffered saline (PBS, Gibco) at 1.9 mg/mL and sterile filtered. It was stored at − 20 °C in aliquots appropriately sized for the individual dosing days. The formulation was brought to room temperature just prior to use. Intratracheal instillation (i.t.) of bleomycin is a commonly used method to create a pulmonary fibrosis model in animals to study diseases including idiopathic pulmonary fibrosis (IPF) [30]. For bleomycin instillation, the rats were anesthetized, via 5% isoflurane inhalation (Meriol, Essex, UK) and positioned head-upwards on a board inclined at a 45° angle. The vocal cords were visualized by passing a paediatric laryngoscope with a plastic cannula into the trachea. Bleomycin (2.5 mg/kg) or vehicle (saline) was then instilled as a 300 µL bolus.

#### Experimental design of in vivo exposure studies

The nose-only inhalation exposure and aerosol characterisation system used were described previously [11] (see the Supplementary Information for further details).

**Fig. 1:**
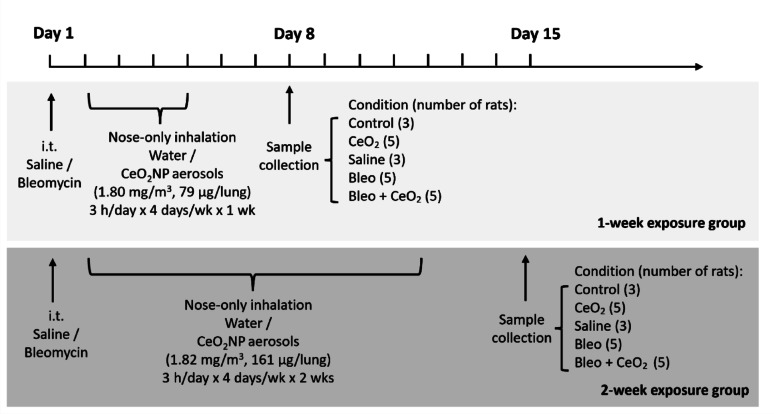
Study design of aerosol exposure experiments using animal models. The rats were randomly allocated to the following five experimental groups: (1) Control: sham exposure to dried H_2_O aerosol (6 animals, 3 per timepoint); (2) CeO_2_: CeO_2_NP aerosol exposure via nose-only inhalation (10 animals, 5 per timepoint); (3) Saline: saline intratracheal instillation followed by nose-only inhalation exposure to dried H_2_O aerosol (6 animals, 3 per timepoint); (4) Bleo: bleomycin intratracheal instillation followed by nose-only inhalation exposure to dried H_2_O aerosol (10 animals, 5 per timepoint); and (5) Bleo + CeO₂: bleomycin intratracheal instillation followed by nose-only inhalation exposure to CeO_2_NP aerosol (10 animals, 5 per timepoint)

The study design is illustrated in Fig. 1. On day 1, all the animals received intratracheal instillations of either bleomycin or saline. From day 2 onwards, they were exposed to aerosolized CeO_2_NPs or water (control) via the nose-only inhalation system for 3 h per day, 4 days per week, over a period of one or two weeks. On days 8 and 15, which corresponded to either 1 or 2 weeks of cumulative exposure to the CeO_2_NP aerosol or control (also corresponding to 1 or 2 weeks following intratracheal instillation of bleomycin or saline), and three days after the final CeO_2_NP aerosol exposure, the animals were euthanized via cardiac puncture under an overdose of intraperitoneal pentobarbital (80 mg/kg). Lung tissues were then harvested, and lung injury was evaluated via BALF analysis, histopathological examination of lung sections, and RNA sequencing of lung tissues. For some of the groups, including the control group and the CeO_2_NP aerosol exposure groups, the same animals were used as in our previous study [11]. As a result, there is some overlap between the groups in this study and those in the earlier publication, and certain analyses of CeO_2_NP aerosol exposure, such as cytological analysis of bronchoalveolar lavage fluid (BALF) and histopathological examination of lung tissue sections, have already been reported in the previous publication.

#### Total cell counts and cell differentiation analysis of bronchoalveolar lavage fluid (BALF)

The rats were sacrificed by exsanguination via cardiac puncture under isoflurane anaesthesia (induced at 5% and maintained at 1.5-2% in 100% oxygen). BALF was collected through a tracheal cannula using two 7 mL aliquots of PBS. The cells from both BALF aliquots were pooled for total and differential cell analysis. The cells were centrifuged at 500 rpm for 5 min and then pelleted onto slides via a cytocentrifuge. The slides were air-dried at room temperature and stained with the Shandon Kwik-Diff Stains Kit (Thermo Scientific). Cell differentials were counted by microscopic examination, with at least 500 cells identified as macrophages, neutrophils, eosinophils, basophils, or lymphocytes on the basis of morphological criteria. The analysis was performed by individuals blinded to the treatment.

#### Lung tissue processing

After bronchoalveolar lavage, the apical and azygous lung lobes were tied off and snap-frozen in liquid nitrogen for transcriptomic analysis. The remaining lung tissue was excised, inflated, and fixed with freshly prepared 4% paraformaldehyde via a tracheal cannula at a pressure of 30 cm in water. The fixed lungs were then processed into paraffin-embedded tissue blocks. Serial 5 μm thick lung sections were cut onto microscope slides for further processing and analysis.

Lung tissue sections were subject to histopathological analysis via two staining protocols: haematoxylin and eosin (H&E) for general histopathology and the Trichrome-Masson method to assess the deposition of collagen, a marker of fibrosis [31].

#### Histopathology analysis

A qualitative assessment of H&E-stained lung tissues from all groups was performed. The analysis was performed by researchers blinded to the treatment groups, and light microscopy was used to examine tissue sections from both the 1-week and 2-week groups. A semiquantitative approach was taken to evaluate the changes in alveolar oedema, the presence of increased numbers of macrophages in alveolar spaces, infiltration of mixed inflammatory cells into alveolar walls and spaces, hyperplasia of bronchiolar club cells at bronchiolar-alveolar junctions, and loss of ciliated cells in the bronchi and bronchioles. The scoring system was as follows: 0 = normal (none), 1 = minimal (occasionally affecting areas comprising up to 5% of the total lung), 2 = mild (affecting areas comprising between 6% and 25% of the total lung), 3 = moderate (frequently affected regions comprising between 21% and 60% of the total lung), and 4 = marked/severe (numerously affected areas composing more than 61% of the total lung).

#### Fibrotic staining analysis

Slides stained with Masson’s trichrome (MT) were examined. To achieve a more comprehensive evaluation of fibrotic alterations across the lung sections, images of entire lung sections were captured. Changes in fibrotic staining were semiquantified as a percentage of the total area via ImageJ software, and the intensity of staining was determined through visualization.

#### RNA sequencing analysis

Lung tissues were homogenized via a bead-based homogenizer (Precellys 24; Stretton Scientific, UK). Aliquots of the lung homogenate were then processed for RNA extraction via the RNeasy Mini Kit and QI shredder (Qiagen, Crawley, UK) following the manufacturer’s protocol. The RNA concentrations were measured via a NanoDrop 1000 spectrophotometer (Thermo Scientific, Waltham, MA, USA). RNA quality was assessed via an Agilent 2100 Bioanalyzer, and only samples with an RNA integrity number (RIN) greater than 8.0 were selected for library preparation. For mRNA sequencing, sample processing and sequencing were performed in collaboration with the Earlham Institute (UK) via the Illumina HiSeq 4000 platform with 150 bp paired-end (150PE) reads. A total of 20 high-throughput stranded RNA libraries were constructed and sequenced in a single lane of the HiSeq 4000 platform, generating 20 million reads per sample.

The raw sequencing data, provided as FASTQ files (one per read direction for each barcoded library), were imported into CLC Genomics Workbench 20.0 (https://digitalinsights.qiagen.com) for RNA sequencing analysis. The paired-end data were mapped to the Rnor_5.0 rat reference genome (Ensembl release 79) with all annotated transcripts extracted via an mRNA track. The reads were aligned to both the transcriptome and the genome via default settings and subsequently categorised and assigned to transcripts via the EM estimation algorithm. Gene expression values were calculated by summing the transcript counts for each gene.

Principal component analysis (PCA) was employed to visualise similarities and differences in gene expression between sample groups. Differentially expressed genes were identified on the basis of a q value threshold of ≤ 0.05 and an absolute fold change ≥ 1.5. In addition, to filter out genes that were not strongly expressed in at least one of the groups being compared, the highest average RPKM (Reads Per Kilobase per Million mapped reads) value for genes across all groups was set to ≥ 5. Gene set enrichment analysis (GSEA) or ingenuity pathway analysis (IPA) were utilized to identify potential pathways involved.

### In vitro/in vivo dose matching

The regional deposition efficiencies and deposited doses for the 1 and 2 week in vivo exposures were estimated from the measured aerosol size distributions, mass concentrations and rat minute ventilation rates via the Multiple-Path Particle Dosimetry (MPPD) model (version 2.11, Applied Research Associates, Inc.) [32]. Further details, including model parameters, can be found in the supplementary information.

The in vitro doses were chosen on the basis of the estimated in vivo doses to the small airways and the dosing limitations imposed by the CeO_2_NP stock suspension concentration and maximum exposure duration, as determined in a preliminary control study to minimize the impact of the exposure system on cell health [33]. The aerosol mass concentration, *C*_*M, ae*_ (µg/cm^3^) required to deliver the target dose, D_A_ (ng.cm^− 2^), in vitro, can then be calculated as

$$\:{C}_{M.ae}=1000.\frac{{D}_{A}A}{Q.t.DE}$$

where A (cm^2^) is the in vitro cell culture insert area, Q (mL.min^− 1^) is the in vitro aerosol delivery flow rate, t is the in vitro exposure duration (min) and DE is the deposition efficiency of the in vitro exposure system (40% from this system/aerosol). Details of the preliminary investigation used to determine the deposition efficiency of the in vitro exposure system are given in the Supplementary Information.

### In vitro aerosol exposure at the air-liquid interface (ALI)

#### Cell culture

Organotypic reconstituted primary human small airway epithelial cell cultures (SmallAir™) were obtained from Epithelix (Geneva, Switzerland). These cultures, which were differentiated and maintained at the air-liquid interface (ALI) on 6.5 mm diameter tissue culture inserts, were kept at 37 °C in a humidified atmosphere with 5% CO_2_. The basolateral media (SmallAir™ culture media # EP65SA) was replaced, and the apical surface was washed every 2–3 days. Cells from five different donors, none of whom had any reported pathologies, were used for experimental exposure. Cultures were exposed to CeO_2_NP aerosols via an aerosol exposure at the air-liquid-interface (AE-ALI) system [33]. Following exposure, the cells were returned to fresh culture conditions for an additional 24 h before samples were collected for gene expression analysis and toxicity assessment.

#### Aerosol exposure and characterization system

A detailed description of the AE-ALI exposure system and its characterisation can be found in Buckley et al. [33]. In brief, CeO_2_NP aerosols were generated from a stock solution via a TSI constant output atomizer (model 3076, TSI Inc., Shoreview, MN, USA). For control exposures, the CeO_2_NP suspension was replaced with Milli-Q water. After generation, the aerosol was dried, diluted via a variable bridge-diluter, conditioned to maintain > 75% RH and 26–28 °C, charge, neutralised and mixed with CO_2_. CO_2_ levels were monitored and adjusted to maintain them at 5 ± 0.2% during exposure.

The conditioned aerosol is then passed to the exposure module (CULTEX^®^ RFS, Cultex^®^ Technology GmbH, Hanover, Germany) and aerosol characterisation instrumentation. The CULTEX RFS is a continuous, perpendicular flow delivery system that delivers aerosols to cells cultured at the air-liquid interface in 3 separate wells [34]. Deposition can be enhanced with the addition of unipolar aerosol charging and electrostatic precipitation. Preliminary system characterization for these CeO_2_NP aerosols revealed that the optimum deposition efficiency and pattern were achieved for 6.5 mm inserts with a minimum achievable electrostatic precipitator voltage of -100 V [33]. To minimize any effects of the system itself on cell health, e.g., from the air flow directed at the cells, an aerosol flow rate of of 5 mL/min to each well was used. In a series of system control exposures to nebulised and dried water in the exposure system described above, it was previously found that cytotoxicity and gene expression increased with increasing exposure duration but limited biological effects were observed for system control exposures of less than 10 min [33]. All exposures were therefore limited to 5 min and different doses were achieved via aerosol dilution.

Aerosol size distributions (SMPS model 3936N76, TSI Inc., Shoreview, MN, USA) were monitored in real-time throughout the exposures, and the average mass concentration was determined gravimetrically using 47 mm glass microfibre filter membranes (Pallflex^®^ Emfab™ filters, TX40HI20WW, Cytiva, Buckinghamshire, UK). Samples for high-resolution TEM imaging (JEOL 3000 F, JEOL Inc. Tokyo, Japan) were taken from the aerosols onto lacey carbon film grids (lacey carbon film 400 mesh copper grids, S166-4, Agar Scientific Ltd., Standsted, Essex, UK) in a Mini Particle Sampler (Ecomesure, Saclay, France), and the deposited particles were captured on solid carbon film grids (carbon film 400 mesh copper TEM grids, AGS16O-4, Agar Scientific Ltd., Stansted, Essex, UK) placed at the bottom of stainless steel insert “dummies” with the same dimensions as the cell culture inserts. The deposited mass (dose) of CeO_2_ was measured via inductively coupled plasma mass spectrometry (ICP-MS), and the spatial distribution of the CeO_2_NPs deposited onto the cells during exposure was visualised using laser ablation ICP-MS. Further details of the methods are given in the Supplementary Information.

#### Experimental design of in vitro exposure studies

**Fig. 2:**
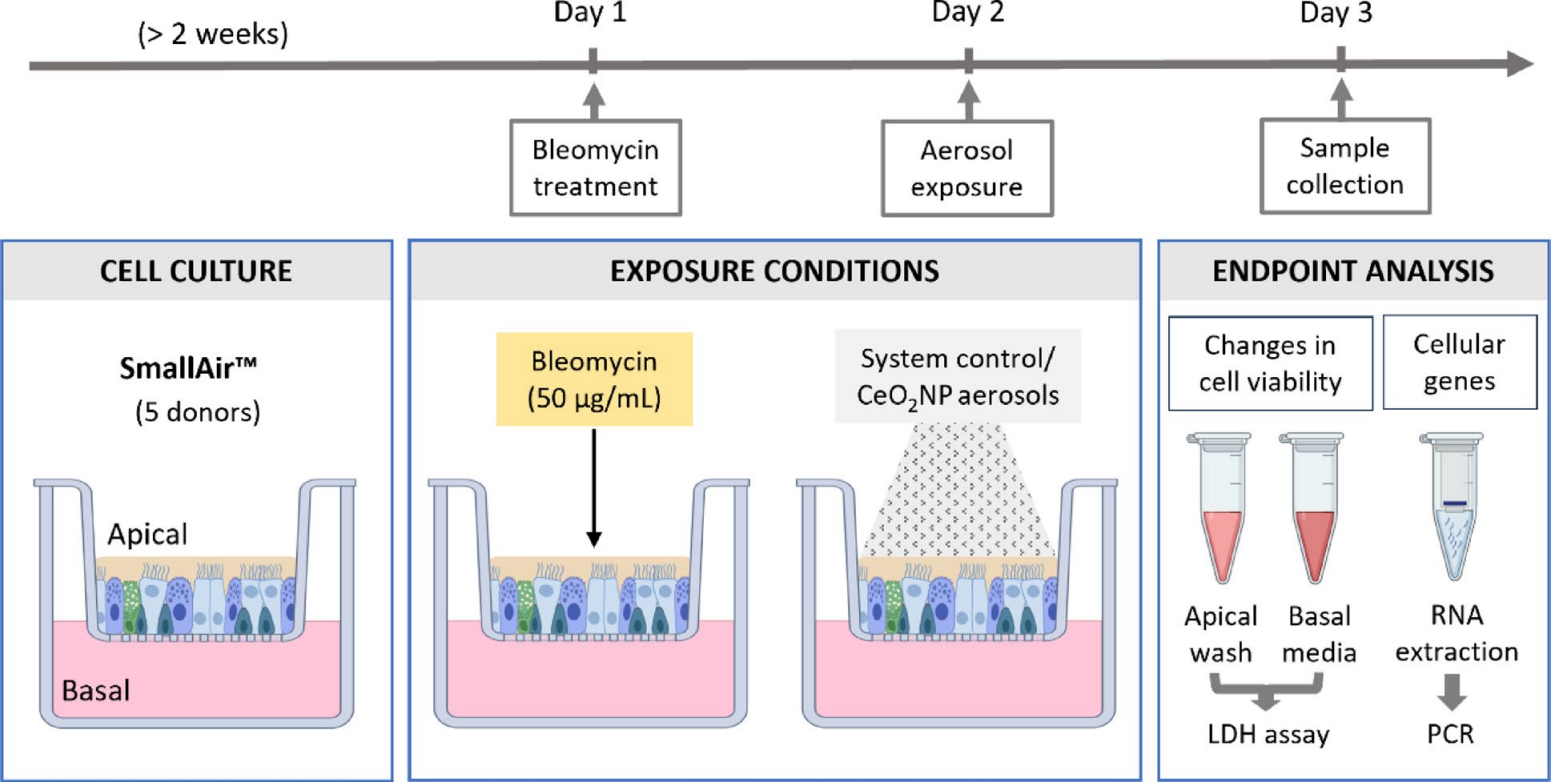
Schematic representation of the in vitro experimental design, including the cell cultures used, bleomycin treatment, CeO_2_NP aerosol exposure at the air-liquid interface (ALI), and subsequent analyses. The control and exposure groups include the IC (incubator control), SC (system control, dried H_2_O aerosol exposure), Bleo (bleomycin + dried H_2_O aerosol exposure), CeO_2__LD (CeO_2_ aerosol exposure at low dose), CeO_2__HD (CeO_2_ aerosol exposure at high dose), Bleo + CeO_2__LD (bleomycin + CeO_2_ aerosol exposure at low dose) and Bleo + CeO_2__HD (bleomycin + CeO_2_ aerosol exposure at high dose) groups

The experimental details are provided in Fig. 2.

A pilot experiment was conducted using the same cellular model to establish conditions for bleomycin treatment (Supplementary Figure S3 & S4). Next, the biological effects of nanosized CeO_2_ aerosol exposure were assessed via the AE-ALI system. A high dose corresponding to the in vivo low dose and a low dose (~ 1/3 of high dose) were applied as described above. Each group underwent three separate consecutive exposure runs. During each run, one well (which differed across the three runs) was used for dose determination, and for each group one TEM sample of deposited particles was collected. Additional characterization runs at high and low doses were conducted to collect samples for laser ablation ICP-MS.

#### Assessment of cell viability

The levels of lactate dehydrogenase (LDH) in the media samples were measured as indicators of membrane integrity and cytotoxicity. The assay was performed via a Promega kit (catalogue no. G1780) according to the manufacturer’s instructions. Briefly, 50 µL of apical wash or basal media was mixed with 50 µL of reconstituted substrate solution and incubated for 30 min at room temperature in the dark. 50 µL of stop solution was then added to each well, and the absorbance was measured at 492 nm.

#### PCR analysis

Total RNA was extracted via the miRNeasy Mini Kit (QIAGEN) following the manufacturer’s protocol. For mRNA analysis, cDNA was synthesised from 1 µg of RNA via the QuantiTect Reverse Transcription Kit (QIAGEN). RT-PCRs were performed in 20 µL volumes containing 1X Fast SYBR^®^ Green Master Mix (Life Technologies), each primer pair (200 nM), and 2 ng of cDNA. The primers were synthesized by Integrated DNA Technologies, and the sequences are listed in Supplementary Table S1. Gene expression data were normalized to human HPRT1 levels via the ΔΔCT method. No amplification signals were observed in the no-template controls. A standard 40-cycle PCR program was used, and fluorescence was detected with the QuantStudio^®^ 6 Flex Real-Time PCR System (Applied Biosystems).

### Statistical analysis

All the data are presented as the means ± standard deviations (SDs) for 3–6 animals (in vivo) or donors (in vitro) per group unless otherwise indicated. Statistical significance was evaluated via a two-tailed Student’s t test unless otherwise stated.

## Results

### Particle characterization

Table 1 summarises the characterisation of the CeO_2_NPs used in in vivo and in vitro exposure respectively. For the in vivo and in vitro stock suspensions, the mean primary particle diameters were determined to be 5.6 ± 1.1 nm and 9.9 ± 1.9 nm (see the Supplementary Information for representative TEM images and further details of the particle sizing), and DLS revealed z-averages (weighted mean hydrodynamic sizes) of 65 nm and 107 nm, suggesting significant agglomeration, confirmed by TEM analysis (Supplementary Figure S1).

**Table 1:** Comparison of CeO_2_NPs, suspensions and aerosols used in the in vivo and in vitro studies

	CeO_2_NP suspension				CeO_2_NP aerosol			
	Suspension Conc.	Mean PP diameter	DLS z-av.	DLS PDI	Mode diameter (CMD)	GSD	Number conc.	Mass conc.
	mg/mL	nm	nm		nm	nm	#/cc x 10^6^	mg/m^3^
In vivo_1 wk	1.0	5.6 ± 1.1	65	0.14	43.4	1.70	2.16	1.80
In vivo_2 wk	1.0	5.6 ± 1.1	65	0.14	43.8	1.72	1.96	1.82
In vitro_LD	3.4	9.9 ± 1.9	107	0.13	87.1	1.71	1.25	7.88
In vitro_HD	3.4	9.9 ± 1.9	107	0.13	85.4	1.72	3.91	28.17

### Aerosol characterization

**Fig. 3:**
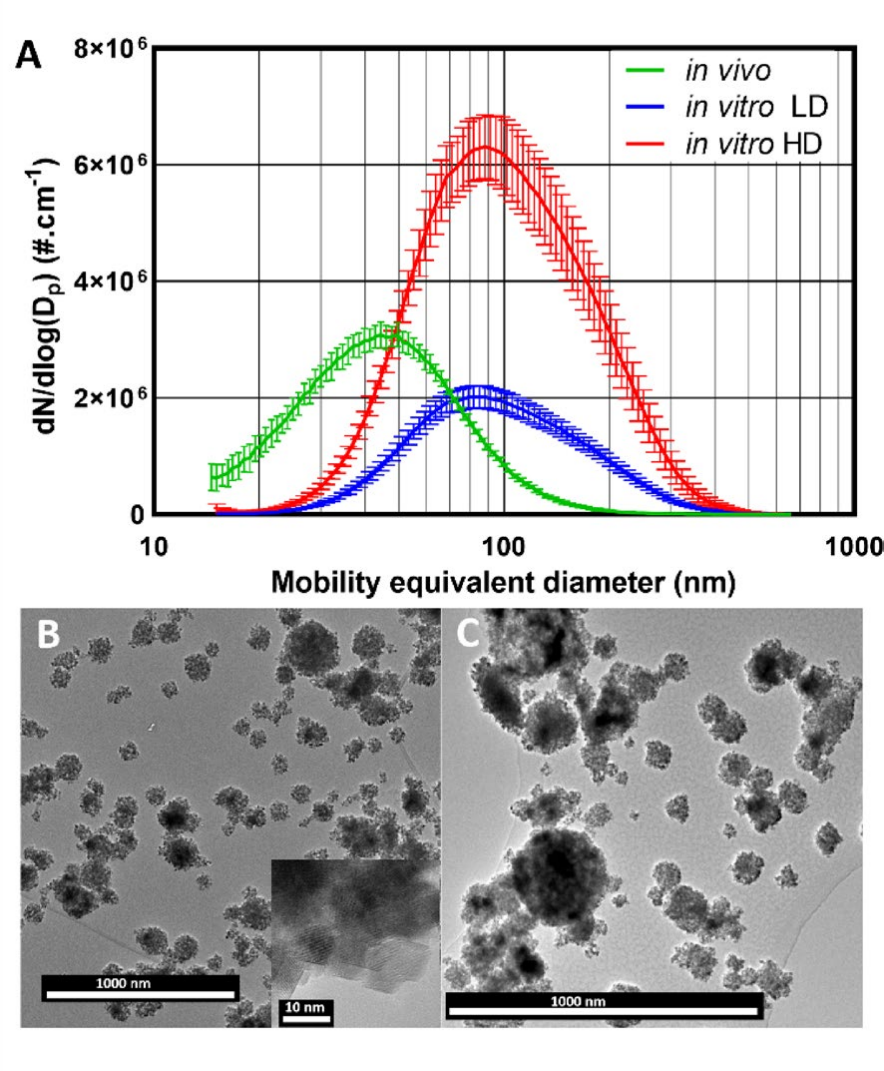
Comparison of the characteristics of aerosols delivered in vivo and in vitro. **A** CeO_2_NP aerosol size distributions delivered in vivo (green) and for the low dose (blue) and high dose (red) in vitro exposure. Representative TEM images of CeO_2_NP aerosols delivered **B** in vitro and **C** in vivo

Aerosol characteristics for the in vivo and in vitro exposures are summarised in Table 1; Fig. 3, including representative images of the agglomerated CeO_2_NP aerosol particles, illustrating their near-spherical form under both exposure conditions, a form typically produced through nebulization and drying of particle suspensions. While the aerosol size distribution was consistent across all in vivo exposure days and all in vitro exposures, owing to the difference in primary particle size and suspension concentrations (Table 1) used to produce the aerosols, there was a significant difference in the distribution of aerosol particle sizes delivered in vivo and in vitro.

### In vivo and in vitro doses

The estimated in vivo doses delivered during the 1- and 2-week in vivo exposures are summarised in Table 2, further details of these calculations are given in the Supplementary Information. Research has demonstrated that small airways are associated with fibrotic and interstitial pulmonary diseases, either as precursors or as exacerbating factors in disease progression [35]. Additionally, our previous inhalation study identified the small airways as the lung region with the highest deposition per unit surface area for nanosized aerosols within the respiratory tract [36]. Therefore, the small airways were selected as the in vitro cellular model for further investigation, and the target doses were chosen to be in line with the in vivo small airway doses. As the CeO_2_NP stock concentration constrained the maximum achievable dose that could be delivered in 5 min, an in vitro dose of 1200 ng/cm^2^ (i.e. matching the in vivo 2-week exposure) was not achievable. Targeted in vitro doses of 600 ng/cm^2^ (i.e. matching the in vivo 1-week exposure) and 200 ng/cm^2^ were therefore chosen, with the lower dose of 200 ng/cm^2^ chosen in part because clearance was not considered in the in vivo dose estimation model. Table 2 also summarises the delivered in vitro doses measured via ICP-MS. The spatial distribution of the CeO_2_NPs deposited onto the cells during in vitro exposure was visualized using laser ablation ICP-MS and TEM (Supplementary Figures S5&S6).

**Table 2:** Summary of mass dose metrics for in vivo and in vitro exposures

	Total dose (µg)	Regional doses (µg)		Mass dose per unit area (ng/cm^2^)	
	Respiratory tract	Small airways	Alveolar region	Small airways	Alveolar region
In vivo_1 wk	79	13	38	629	11
In vivo_2 wks	161	25	76	1258	22
In vitro_LD		-	-	275 ± 77	-
In vitro_HD		-	-	706 ± 151	-

Total in vivo doses were estimated from measured aerosol size distributions, concentrations, and breathing parameters via the MPPD model [32] and converted to doses per unit area assuming areas of 3400 cm^2^ for the alveolar region and 20 cm^2^ for small airways (generations 9–15) [32]. In vitro doses were quantified via ICP-MS and converted to doses per unit area using a cell culture insert surface area of 0.33 cm^2^. Standard deviations (±) are reported for in vitro doses, which are experimentally measured, whereas they are not provided for in vivo estimates, as they are derived from model predictions.

### Biological endpoint analysis of the in vivo exposure study

#### Cytological changes induced by the inhalation of CeO₂NPs with bleomycin pretreatment

**Fig. 4:**
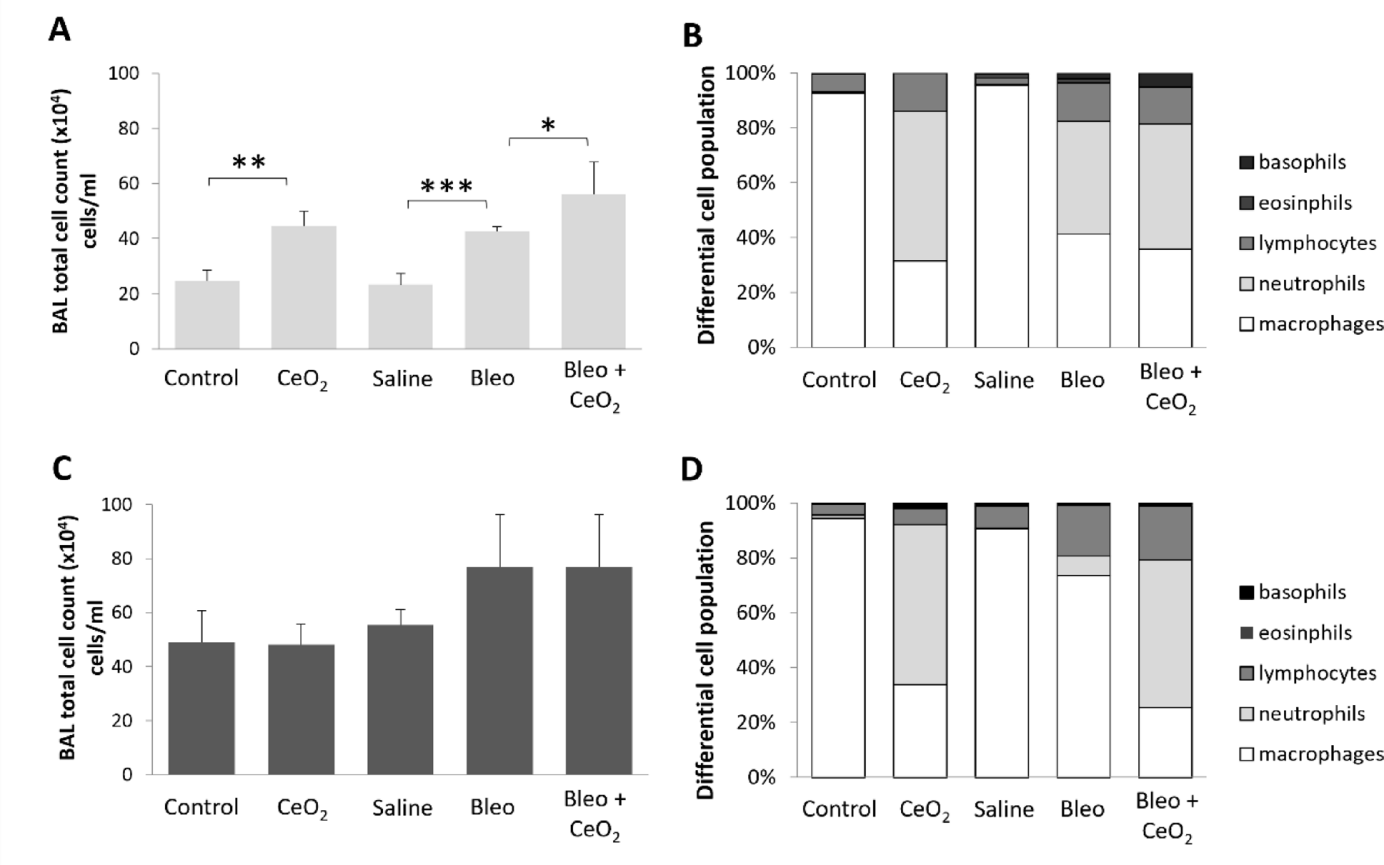
Cytological analysis of BALF. Comparison of changes in the total cell count (**A**, **C**) and differential cell population (**C**, **D**) in the BALF after the inhalation of CeO_2_NP or control aerosols without and with bleomycin pretreatment (A and B, 1 week of exposure to CeO_2_NP aerosols; C and D, 2 weeks of exposure to CeO_2_NP aerosols). The data are shown as means ± SDs (*n* = 5 rats for the Bleo-treated and/or CeO_2_-exposed groups and *n* = 3 for the control and saline groups). * *P* < 0.05, ** *P* < 0.01, *** *P* < 0.001

The total and differential cell counts in bronchoalveolar lavage fluid (BALF) for each group are presented in Fig. 4. In the 1-week exposure group (Fig. 4A), the total cell count was significantly greater in the CeO_2_NP-exposed rats than in the aerosol control group (*P* < 0.01) and was similarly higher in the bleomycin-treated rats than in the saline-treated rats (*P* < 0.001). The total cell count in the group receiving bleomycin treatment followed by CeO_2_NP-exposure was even greater than that in the bleomycin-only treated group. In the 2-week exposure group (Fig. 4C), no significant differences were observed among any of the conditions.

In the 1-week exposure group, the percentage of neutrophils was significantly greater in bleomycin-treated rats than in saline-treated rats, whereas this difference was not observed in the 2-week exposure group (Fig. 4B and D). Analysis of absolute cell numbers supported these findings (Supplementary Figure S7). In the 1-week exposure group, changes in the absolute number of macrophages were not significant, but in the 2-week exposure group, there was a significant decrease in the absolute number of macrophages in rats treated with bleomycin followed by CeO_2_NP exposure (199,348 ± 34,474) compared with those treated with bleomycin alone (563,549 ± 59,952). In contrast, the absolute number of neutrophils was significantly greater in the bleomycin + CeO_2_NP group (410,585 ± 82,885) than in the bleomycin alone group (58,792 ± 21,061). A comparable neutrophil influx was observed in the CeO_2_NP only group, indicating that this response was independent of bleomycin pretreatment and suggesting that neutrophil infiltration was primarily induced by inhaled CeO_2_NPs. No statistically significant differences were observed in the basophil, eosinophil or lymphocyte counts across the treatment groups.

#### Inflammatory alterations in lung tissues observed via histopathological examination

**Fig. 5:**
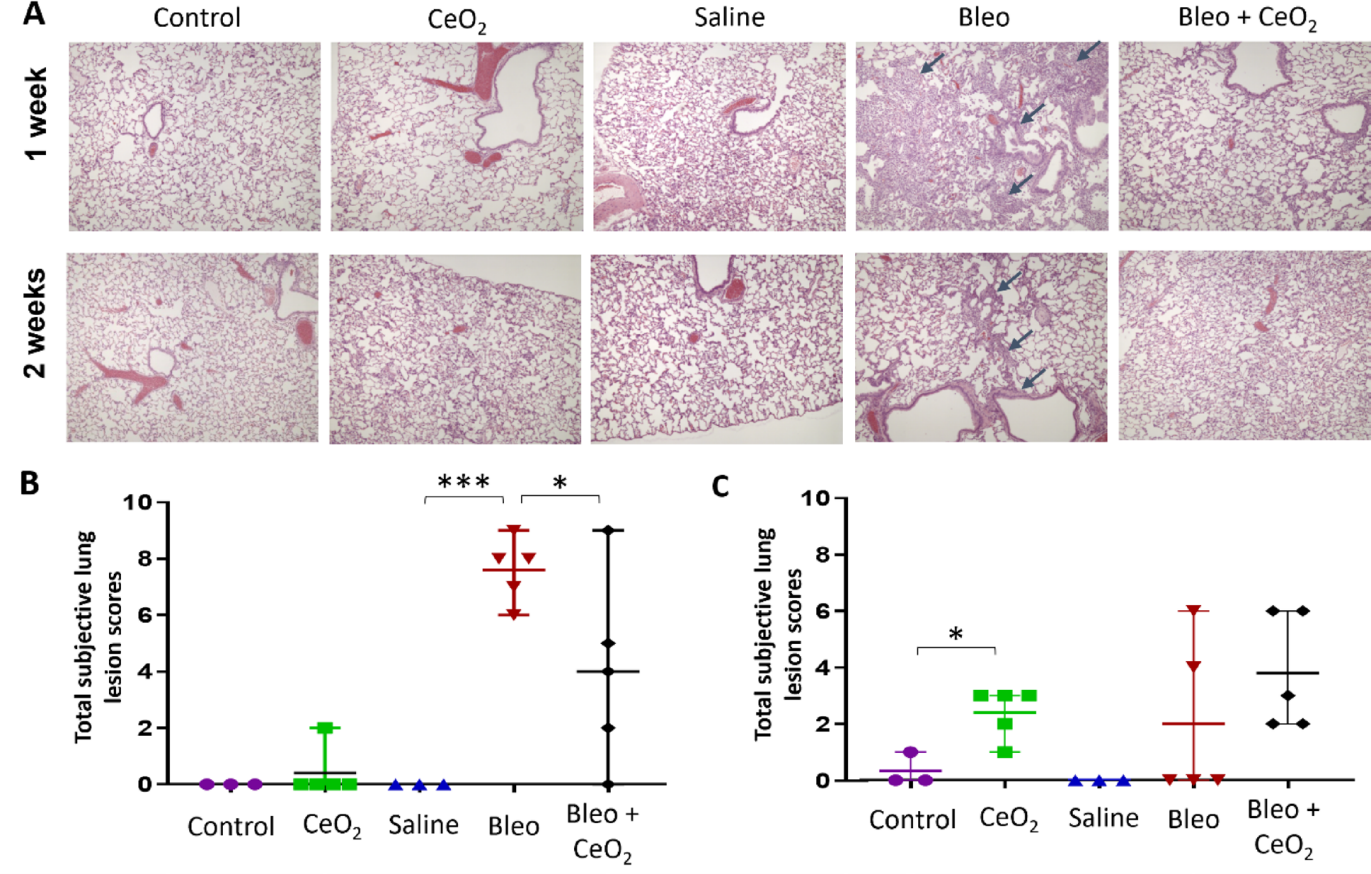
Histopathological examination of lung tissue sections. **A** Representative hematoxylin and eosin (H&E) -stained lung sections (10X) from rats in different control and exposure groups. The arrows indicate representative areas of observed histopathological changes. **B**, **C** Total subjective lung lesion scores for individual animals after 1 week (**B**) or 2 weeks (**C**) of exposure to CeO_2_NPs or control aerosols without or with bleomycin pretreatment. Lines represent the means and ranges. The data are shown as means with each individual value for each animal (*n* = 5 rats per group for the exposed group and *n* = 3 for the control group). * *P* < 0.05, *** *P* < 0.001

The lung lesions observed in haematoxylin and eosin (H&E)-stained lung sections were evaluated (Fig. 5). Consistent with the results of BAL analysis, bleomycin treatment elicited a robust inflammatory response, with significant histopathological changes in the lung tissue. These included a marked increase in patchy areas of alveolar damage, characterized by mixed inflammatory cell infiltration within the alveolar walls, the presence of spindle-shaped cells (likely fibroblasts), and frequent cuboidal cell lining, indicative of type II alveolar hyperplasia. In these areas, alveolar spaces are filled with mixed inflammatory cells, primarily macrophages and lymphocytes, accompanied by variable oedema. In some areas, the alveolar structure was completely obliterated. At the bronchiolar-alveolar junctions, non-ciliated epithelial cells (club cells) exhibit hyperplasia with pronounced disorganization, occasionally extending into regions of damaged alveolar walls, which is indicative of alveolar bronchiolization.

The severity of these changes decreased markedly over time, with the number of lung lesions substantially lower at 14 days (2-week exposure condition) than at 7 days (1-week exposure condition) post-bleomycin administration, particularly in the alveolar and bronchial regions. There was a notable decrease in the number of macrophages within alveolar spaces and a reduced infiltration of mixed inflammatory cells into alveolar walls and spaces. Additionally, hyperplasia of bronchiolar club cells at the bronchiolar-alveolar junctions was significantly less pronounced.

To evaluate the effects of inhaled CeO_2_NP aerosol particles on bleomycin-induced pulmonary injury, a model for active disease processes, a comparison was conducted between rats treated with bleomycin alone and those pretreated with bleomycin followed by CeO_2_NP exposure. In the 1-week exposure group, the subjectively assessed total lung scores were significantly greater in the bleomycin-treated rats than in the saline-treated rats, (and also significantly greater than the rats exposed to control and CeO_2_NP aerosols). The severity of lung lesions in rats pretreated with bleomycin followed by CeO_2_NP exposure was significantly attenuated (*P* < 0.05), with a wide range of scores, as shown in Fig. 5B. In the 2-week exposure group (Fig. 5C), the differences in the severity of lung lesions among the various exposure conditions were minimal, with less pronounced distinctions observed.

#### Analysis of fibrotic alterations in lung tissue sections

**Fig. 6:**
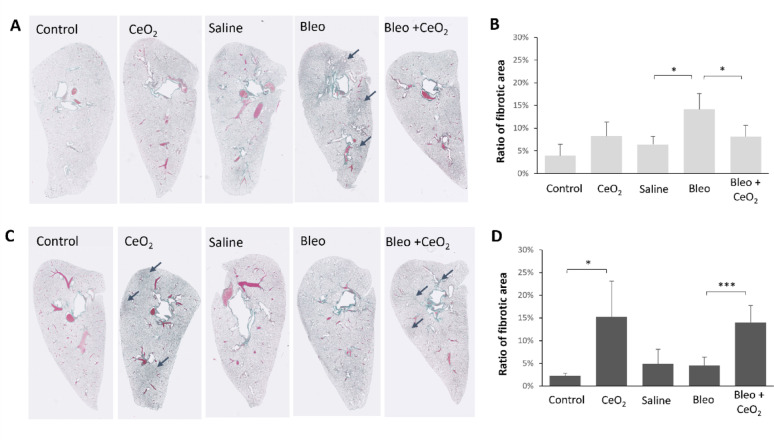
Analysis of fibrotic staining in lung tissue sections. **A**, **C** Representative Masson’s trichrome-stained whole-lung sections from rats in different control and exposure groups. The arrows indicate representative fibrotic regions identified by blue/green staining. **B**, **D** Ratios of the fibrotic area to total lung area in individual animals after 1 week (**B**) or 2 weeks (**D**) of exposure to CeO_2_NPs or control aerosols without or with bleomycin pretreatment. The data are shown as the means ± SDs (*n* = 5 rats for the Bleo-treated and/or CeO_2_-exposed groups and *n* = 3 for the control and saline groups). * *P* < 0.05, *** *P* < 0.001

Fibrotic alterations between the conditions were assessed by measuring the percentage of the fibrotic area (Fig. 6). Consistent with the histopathological findings, in the 1-week exposure group, bleomycin pretreatment significantly increased the percentage of fibrotic staining in lung tissue sections from rats treated with bleomycin alone, reaching approximately 15%. In contrast, fibrotic staining was markedly reduced in rats treated with bleomycin followed by CeO_2_NP exposure. In the 2-week exposure group, the changes in fibrotic staining induced by bleomycin were reversed at 14 days (2-week exposure condition) compared with 7 days (1-week exposure condition) post-bleomycin administration. However, CeO_2_NP exposure led to a significant increase in the percentage of fibrotic staining, regardless of bleomycin treatment.

When the fibrotic alterations induced by bleomycin and CeO_2_NP exposure were compared, the areas affected by bleomycin were predominantly localized near the bronchi, whereas inhaled CeO_2_NPs resulted in a more homogeneous hyperplasia throughout the lung (Supplementary Figure S8). This difference may be attributed to the distinct routes of administration: bleomycin was administered via intratracheal instillation, leading to localized effects in specific regions of the lung, particularly in areas around bronchial airways, whereas CeO_2_NPs were more uniformly distributed and deposited throughout the lung tissue.

#### Transcriptomic profiles of lung tissues following the inhalation of CeO2NPs with prior bleomycin pretreatment

**Fig. 7:**
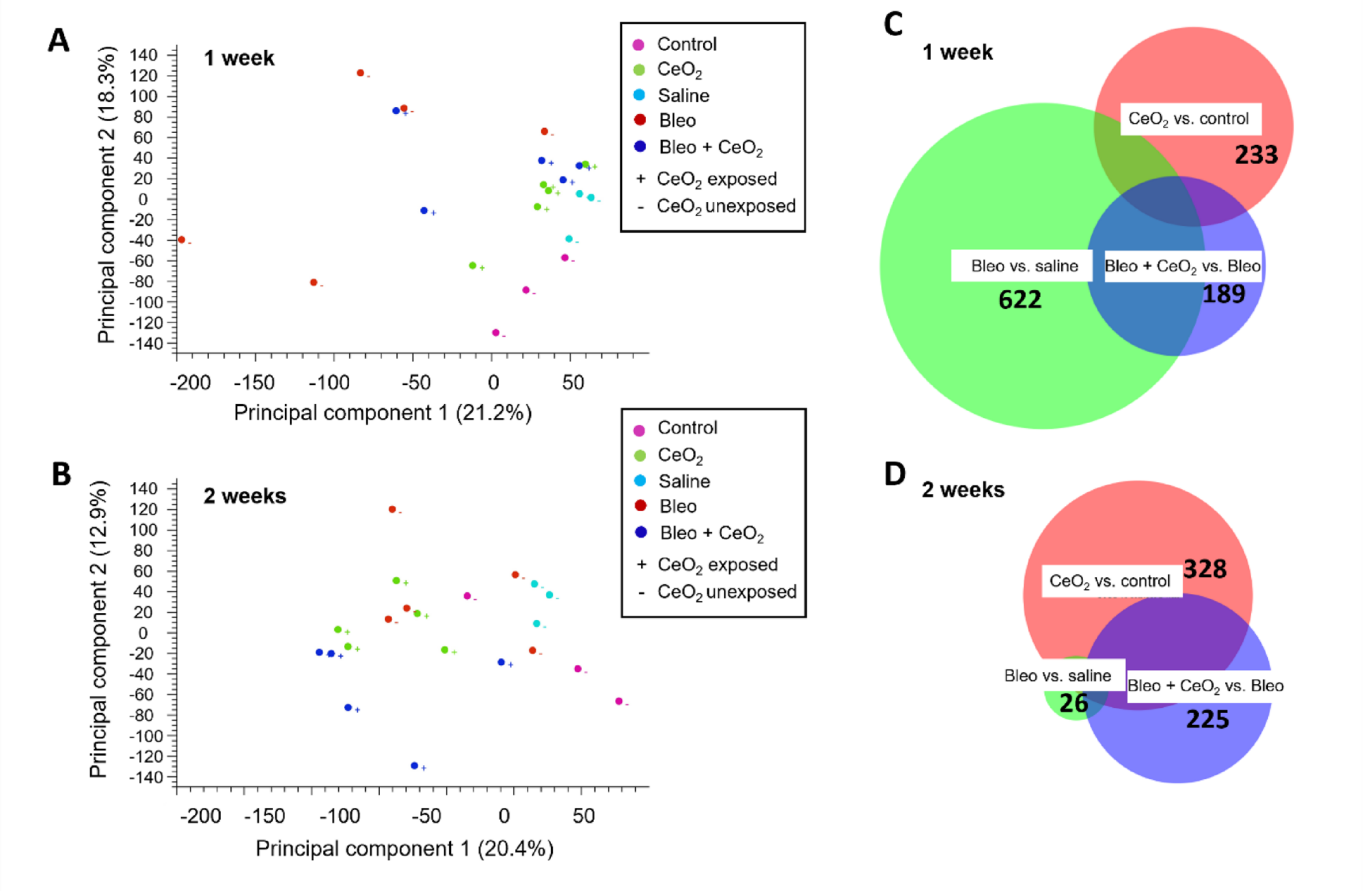
RNA sequencing analysis. **A**, **B** Principal component plots. **A** Groups after 1 week of exposure to CeO_2_NPs or control aerosols. **B** Groups exposed to CeO_2_NP or control aerosols for 2 weeks. **C**, **D** Venn diagram of differential expressed genes (DEGs) in the **A** groups after 1 week exposure to CeO_2_NP or control aerosols, and **B** groups after 2 weeks of exposure to CeO_2_NP or control aerosols. A total of 32,623 variables were included in the analysis under statistical parameters, with a minimum “max group mean RPKM” of 5, a minimum absolute fold change of 1.5 and a maximum FDR of 0.05

As demonstrated above, a single administration of bleomycin via intratracheal instillation induced pulmonary fibrotic effects after 7 days, characterised by an early inflammatory phase. Notably, the effects were largely resolved by 14 days. In addition, exposure to CeO_2_NPs reduced both the histopathological severity and fibrotic changes observed at 7 days post-bleomycin treatment. Consequently, more sensitive and high-throughput methods, including transcriptomics, have been utilized to gain a deeper understanding of the molecular mechanisms triggered by exposure to CeO_2_NPs, particularly in the context of bleomycin pretreatment (Fig. 7). The principal component analysis (PCA) score plot from the transcriptomic analysis of the 1-week and 2-week exposure groups revealed a clear separation of CeO_2_NP exposure from control aerosol exposure (Supplementary Figure S9A). The longer 2-week exposure resulted in greater deposition of CeO_2_NPs in the lungs, leading to more significant perturbations in gene transcription than the shorter 1-week exposure. Transcriptomic profiling also revealed the effects of bleomycin treatment in inducing pulmonary injury and active disease processes (Supplementary Figure S9B), with a clear separation between bleomycin treatment and saline instillation conditions. Notably, the separation observed at 7 days post-bleomycin administration (1-week exposure group) appeared to be more significant than that at 14 days post-bleomycin administration (2-week exposure group).

Transcriptomic profiling was employed to assess the effects of inhaled CeO_2_NPs on bleomycin-induced pulmonary injury. The analysis of the 1-week exposure group demonstrated a clear separation between the bleomycin treatment and all other conditions, with the bleomycin + CeO_2_NP condition positioned between the bleomycin treatment and the remaining conditions (Fig. 7A). In contrast, the separation among the various conditions in the 2-week exposure group was not significant (Fig. 7B).

Further statistical analysis via the transcriptomic profiling was performed to identify the differentially expressed genes (DEGs) associated with the fibrotic effects induced by bleomycin treatment and the subsequent effects triggered by CeO_2_NP aerosol exposure (Fig. 7C and D). With a minimum “max group mean RPKM” of 5, a fold change cut-off of 1.5 and a maximum false discovery rate (FDR) of 0.05, there were 622 DEGs at 7 days post bleomycin administration in comparison with those in the saline instillation group (1-week exposure group), but the number of DEGs was much lower at 14 days post bleomycin administration (2-week exposure group). The DEGs reflecting the effects of inhaled CeO_2_NPs on bleomycin-induced pulmonary injury were identified by comparing the bleomycin + CeO_2_NP condition with the bleomycin-only condition. There were 189 DEGs in the 1-week exposure group and 225 DEGs in the 2-week exposure group. Notably, in the 2-week exposure group, approximately 50% of the overlapping genes were regulated by CeO_2_NP exposure under conditions with and without bleomycin treatment.

The set of 189 DEGs identified by comparing the bleomycin + CeO_2_NP condition with the bleomycin-only condition was input for gene set enrichment analysis (GSEA) to elucidate the underlying biological processes influenced by CeO_2_NPs in the context of bleomycin pretreatment. The epithelial-mesenchymal transition (EMT) pathway was identified as being inhibited by CeO_2_NP exposure. EMT is a reversible process in which epithelial cells lose their polarity and cell-cell adhesion ability while acquiring migratory and invasive capabilities. Changes in the expression of several genes implicated in EMT were examined under various exposure conditions, as shown in Supplementary Figure S10. These genes include Fn1 (fibronectin-1), Fndc1 (fibronectin type III domain containing 1), TGFβ-3 (transforming growth factor beta-3), and Col12a1 (alpha chain of type XII collagen), along with fibrosis markers such as Spp1 (osteopontin) and Timp1 (tissue inhibitor of matrix metalloproteinase-1). In the 1-week exposure group, most of these genes were significantly upregulated following bleomycin treatment; however, this upregulation was reversed when CeO_2_NP exposure followed bleomycin administration. In contrast, in the 2-week exposure group, no statistically significant changes were observed for most of these genes, except for Spp1 and Timp1, whose expression was increased in the rats treated with bleomycin followed by CeO_2_NP exposure compared with the other groups.

An additional approach to transcriptomic pathway analysis was carried out via a commercially available database (Ingenuity Pathway Analysis (IPA)) (details in the Supplementary Information) to provide a more comprehensive assessment of how CeO_2_NP exposure modifies the biological processes altered by bleomycin exposure (Supplementary Figure S11). The activation of DNA damage pathways was identified with bleomycin alone at 1-week of exposure, which is consistent with the molecular mechanism of bleomycin-induced cellular toxicity [37]. This DNA damage response was absent when the animals were subsequently exposed to CeO_2_NPs for 1 week. At 2 weeks after bleomycin administration, there were no significant alterations in pathway activation. However, bleomycin treatment followed by 2 weeks of CeO_2_NP exposure resulted in predominant inflammatory and immune cell activation pathway activation (Supplementary Figure S11).

### Adverse effects of CeO_2_NP aerosols on bleomycin pretreated human airway epithelium models via aerosol exposure at the air-liquid-interface (AE-ALI)

Previously, we conducted a detailed characterisation of the AE-ALI system [33]. Our findings revealed that air exposure alone can significantly impact cells, including inducing cytotoxicity and altering the expression of genes such as CXCL1, HMOX1, and SPP1, even at relatively short exposure durations (over 10 min). In the present study, exposure to a water aerosol (the suspending medium of the CeO_2_NPs) was included as a system control alongside the incubator control, to evaluate potential exposure system-related effects.

**Fig. 8:**
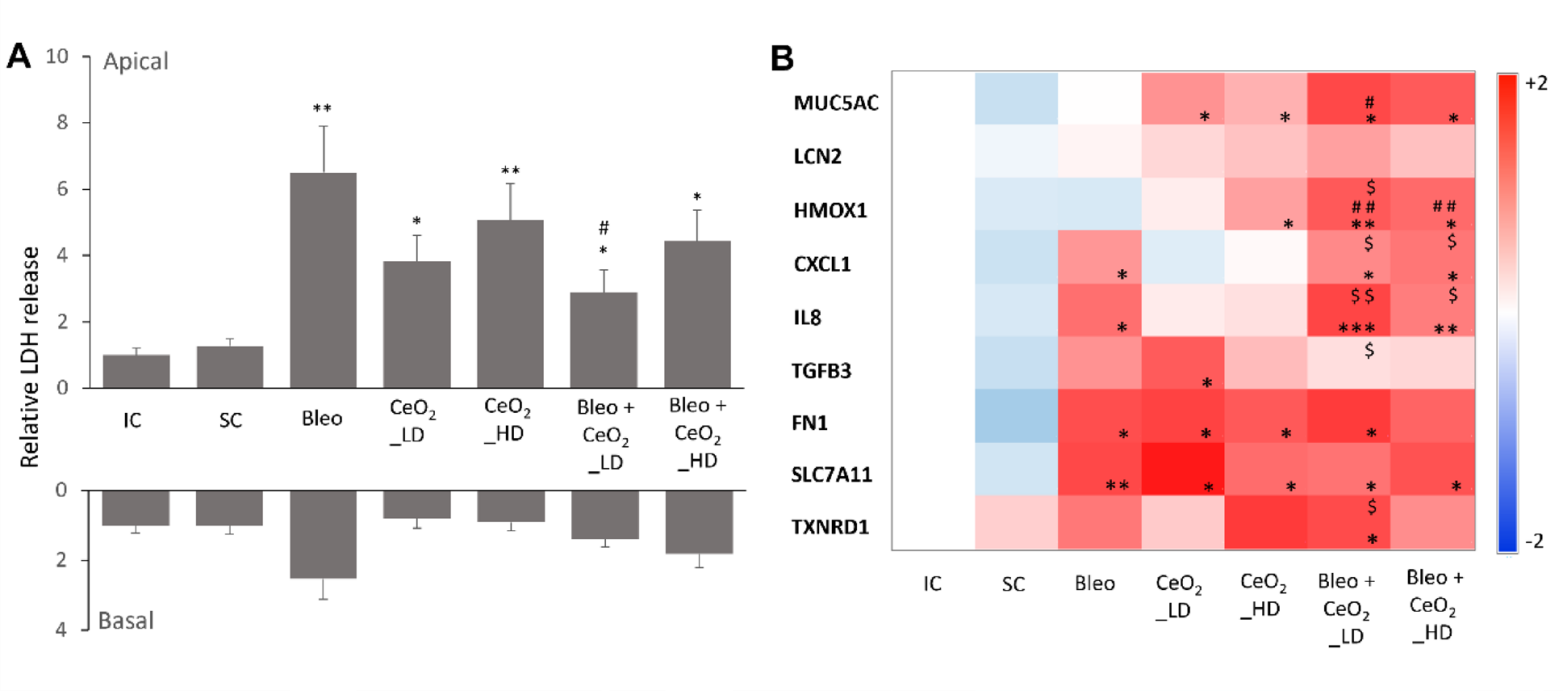
Toxicity assessment of nanosized CeO_2_NP aerosol particles at the air-liquid interface with prior bleomycin treatment. **A** Cytotoxicity analysis of SmallAir™ at 1 day following various exposure conditions (IC: incubator control; SC: system control – dried H_2_O aerosol exposure; Bleo: bleomycin + dried H_2_O aerosol exposure; CeO_2_NP_LD: CeO_2_NP aerosol exposure (low − 275 ± 77 ng/cm^2^); CeO_2_NP_HD: CeO_2_NP aerosol exposure (high − 706 ± 151 ng/cm^2^); Bleo + CeO_2_NP_LD : bleomycin + CeO_2_NP aerosol exposure (low); Bleo + CeO_2_NP_HD: bleomycin + CeO_2_NP aerosol exposure (high)). The relative LDH release in the apical and basal media was normalized to that of the individual donor incubator control. The data are shown as means ± SDs. Compared with the SC condition (system control), statistical significance was observed (* *p* < 0.05, ** *p* < 0.01). Compared with Bleo + H_2_O (bleomycin + H_2_O aerosol exposure), statistical significance was observed (# *p* < 0.05). **B** Heatmap displaying the fold change in the expression of selected genes in SmallAir™ at 1 day following various exposures. Gene expression alterations were normalised to those of the respective controls from individual donors. Statistical significance was assessed via paired t-tests. Compared with the system control condition, the difference was statistically significant (* *p* < 0.05, ** *p* < 0.01, *** *p* < 0.001). Compared with the condition of Bleo (bleomycin + H_2_O aerosol exposure), the difference was statistically significant (# *p* < 0.05, ## *p* < 0.01). Compared with respective CeO2 aerosol exposure groups, the difference was statistically significant ($ *p* < 0.05, $$ *p* < 0.01)

LDH release was measured 24 h postexposure in both the apical and basal media to evaluate cytotoxicity (Fig. 8A and Supplementary Figure S12). Control aerosol exposure alone did not induce significant changes in LDH release in either the apical or basal media. However, both H₂O aerosol exposure following bleomycin pretreatment and CeO_2_NP aerosol exposure alone significantly increased LDH levels in the apical culture medium, reflecting increased plasma membrane permeability. LDH release on the apical side was notably greater at both high and low exposure dose of CeO_2_NP compared with the control groups, indicating that CeO_2_NPs compromised cell membrane integrity. LDH release on the basal side was not significantly different across these groups. Under bleomycin pretreatment conditions, CeO_2_NP exposure significantly increased LDH release on the apical side compared with that in both the incubator and system control groups, with a similar magnitude of increase and dose-dependent trend as those observed with CeO_2_NP exposure alone. Notably, compared with bleomycin pretreatment alone, a low exposure dose of CeO_2_NPs significantly reduced LDH release on the apical side, whereas a high exposure dose did not significantly change LDH release. LDH release on the basal side remained unaffected across all conditions. These results suggest that CeO_2_NP exposure elicits dose-dependent effects on bleomycin-induced cellular responses.

Gene expression analysis was conducted to evaluate the biological responses of SmallAir™ cultures under various exposure conditions (Fig. 8B and Supplementary Figure S13). The tested genes included small airway-specific markers (e.g., MUC5AC), markers of oxidative stress and inflammation (e.g., HMOX1, LCN2, CXCL1, IL-8, SLC7A11, and TXNRD1), and selected genes relevant to EMT (e.g., FN1 and TGFB3). The system control did not result in significant alterations in the expression of the tested genes. In contrast, bleomycin treatment followed by control aerosol exposure significantly upregulated several genes, particularly those involved in inflammation (e.g., CXCL1 and IL-8), oxidative stress (e.g., SLC7A11), and fibronectin (FN1) levels. Compared with bleomycin treatment, exposure to CeO_2_NPs in the AE-ALI system induced relatively moderate changes in gene expression. Exposure to CeO_2_NPs alone resulted in variable but notable effects on gene expression, including significant upregulation of genes such as MUC5AC, TGFB3, and FN1.

Compared with bleomycin pretreatment alone, CeO_2_NP exposure after bleomycin pretreatment led to increased expression of genes such as MUC5AC and HMOX1. Compared with those exposure to CeO_2_NPs alone, the expression of specific genes, including CXCL1, HMOX1, IL-8 and TXNRD1 (low dose only), was increased, whereas the expression of other genes was not significantly changed. Notably, the expression of TGFB3 was significantly reduced at low doses of CeO_2_NP exposure following bleomycin pretreatment. These findings suggest that CeO_2_NPs have dose- and context-dependent effects on gene expression, potentially by modulating bleomycin-induced responses.

## Discussion

### Modulatory effects of CeO_2_NPs on bleomycin-induced inflammation and fibrotic events in in vivo models

In this study, the exposure of Sprague-Dawley rat to CeO_2_NPs alone elicited inflammatory responses as presented in a previous publication [11]. As anticipated, bleomycin treatment resulted in elevated total bronchoalveolar lavage (BAL) cell counts, increased fibrotic lung staining, and increased expression of genes linked to inflammation and oxidative stress. Bleomycin-induced lung injury models were used to investigate the effects of inhaled CeO_2_NPs on active disease processes relevant to human fibrotic lung conditions. The deposited doses of CeO_2_NPs (approximately 79 µg per lung following one week of exposure and 161 µg per lung after two weeks of exposure), although exceeding previously reported estimates of human lifetime exposure [2, 7], were comparable to them, in line with the study’s objective to support a mechanistic investigation under real world conditions. CeO_2_NP exposure began 24 h after bleomycin administration in both the one-week and two-week groups, with the animals being sacrificed three days after the final CeO_2_NP exposure. This approach allows the assessment of CeO_2_NP effects during the “active” phase of disease processes underlying fibrotic mechanisms. However, the overall post-bleomycin endpoint analysis time points differed: seven days in the one-week exposure group and fourteen days in the two-week group. These treatment groups therefore reflect two variables simultaneously: the CeO_2_NP exposure dose and the timing of endpoint analysis post- bleomycin administration.

Our findings demonstrated that exposure to CeO_2_NPs modulated bleomycin-induced responses in the one-week exposure group, as evidenced by reduced fibrotic staining and changes in the expression of genes associated with lung function, inflammation, and epithelial-mesenchymal transition (EMT). These results are consistent with a previous study employing CeO_2_NPs as a delivery system for microRNA-146a in the localized treatment of acute lung injury, where CeO_2_NPs alone could attenuate the fibrotic effects, including reductions in inflammatory cell infiltration, reactive oxygen species (ROS) levels, and collagen deposition post-bleomycin injury [24]. Bleomycin-induced lung injury typically progresses from an acute inflammatory phase marked by neutrophil infiltration to a fibrotic phase characterised by excessive collagen and extracellular matrix deposition. The protective effects observed after one week of CeO_2_NP exposure may depend on both dose and timing. If the sacrifice time point for this group had been extended without additional weeks of continued CeO_2_NP exposure, it would be difficult to predict whether the protective effects would be sustained. On the basis of the extent of development in fibrotic staining and the observed changes in DEGs, our findings suggest that seven days post-bleomycin instillation represents an optimal time point for evaluating lung fibrotic alterations. This earlier assessment time differs from the recommendations of other studies [38], with variability potentially attributable to differences in instillation dose, bleomycin source, administration frequency, and other experimental factors.

The modulatory effects were not significant in the two-week exposure group in the present study, likely because of differences in toxicity at distinct time points following bleomycin administration. Compared with those at seven days post-bleomycin, the toxic effects of bleomycin at fourteen days post administration appeared to be reduced, as indicated by milder histopathological alterations, reduced fibrotic responses, and fewer DEGs observed. These results suggest a partial recovery from bleomycin-induced pulmonary toxicity. This time-dependent attenuation of fibrotic staining aligns with previous reports describing the transient nature of bleomycin-induced pulmonary toxicity [38–40]. Unlike bleomycin, the increased fibrotic responses induced by inhaled CeO_2_NPs were broadly dose- and time dependent, with pulmonary toxicity progressively increasing over time. The fibrotic effects worsened between 3 and 7 days postexposure, as demonstrated in our previous inhalation study [11]. Therefore, in the two-week exposure group, the administration of CeO_2_NPs to already established fibrotic tissue may predominantly reflect nanoparticle-specific effects. These findings suggest that bleomycin and CeO_2_NPs induce lung injury through distinct biological mechanisms. In addition, differences in the exposure route, instillation versus inhalation, likely contribute to the distinct spatial distribution of fibrotic lesions within the lung. These factors may together explain the differing outcomes across the one-week (low-dose) and two-week (high-dose) exposure groups in this study.

### Potential underlying mechanisms

#### Toxicological effects of bleomycin

Bleomycin, a group of compounds derived from *Streptomyces verticillus*, is widely used as a chemical drug but is well recognised for its dose-limiting side effect on pulmonary fibrosis. At the molecular level, bleomycin-induced toxicity is driven by its bithiazole moiety, which intercalates into the DNA helix, together with pyrimidine and imidazole groups that coordinate with iron and oxygen. This coordination forms an activated complex (O₂-Fe(II)-bleomycin), which generates reactive oxygen species (ROS) near the DNA polynucleotide chains, causing localized oxidative damage, strand breaks, and eventual DNA degradation [41]. Consistent with this mechanism, bleomycin exposure in our study induced pronounced transcriptomic alterations that amplified inflammatory and fibrotic signalling cascades. Notably, FN1, encoding fibronectin, was significantly upregulated in both in vivo and in vitro experiments, in agreement with previous reports linking bleomycin-induced oxidative stress to extracellular matrix remodelling and fibrotic progression [42, 43].

#### Oxidative stress and protective mechanisms of CeO_2_NPs

CeO_2_NPs are known to exhibit both pro-oxidant and antioxidant properties, depending on the context. On the one hand, CeO_2_NPs can be internalized by cells and increase the intracellular levels of reactive oxygen species (ROS), leading to oxidative stress and potential cellular damage [44]. This pro-oxidant activity is influenced by the cerium oxidation state present on the nanoparticle surface. While cerium is predominantly in the Ce^4+^ oxidation state, surface defects result in the presence of Ce^3+^ on the particle surface. Notably, for very small CeO_2_NPs (3–30 nm), the majority of the surface cerium exists as Ce³⁺ [45]. Consequently, alterations in the Ce^3+^/Ce⁴⁺ ratio on the nanoparticle surface following exposure can contribute to the cytotoxic effects [11, 46]. On the other hand, the antioxidant properties of CeO_2_ and other nanoparticles are thought to arise from their ability to function as free radical scavengers, providing protection against chemical, biological, and radiological insults that promote free radical generation [47, 48]. The redox cycling between Ce^3+^ and Ce^4+^ underlies the unique antioxidant chemistry of engineered CeO_2_NPs, supporting their potential as safe and effective biological antioxidants. This finding was confirmed in a study in which CeO_2_NPs were used as a delivery system for microRNA-146a in animal models. CeO_2_NPs alone were able to reduce CM• nitroxide production to control levels and significantly attenuated leukocyte recruitment, accompanied by a decrease in inflammatory signalling, including a marked reduction in IL-6 expression in bleomycin-induced inflammation [24]. The redox ability of CeO_2_NPs suggests that oxidative mechanisms contribute to their modulatory effects on bleomycin-induced active pulmonary disease processes. This finding is supported by the expression of some oxidative stress-relevant genes in the in vivo RNA-sequencing analysis, such as HMOX1 expression (data not shown), which was upregulated following bleomycin treatment in the one-week exposure group; however, its expression was significantly reduced after CeO_2_NP exposure. HMOX1 encodes heme oxygenase-1, a key enzyme in the cellular adaptive response to oxidative stress. These findings align with previous studies showing that CeO_2_NPs can modulate ROS levels [27], highlighting their role in reducing oxidative stress and influencing downstream fibrotic mechanisms.

#### Modulatory effects of CeO_2_NPs on bleomycin-induced active pulmonary disease processes

The RNA sequencing results indicated that one-week exposure to CeO_2_NPs reduced bleomycin-induced fibrotic alterations possibly through inhibiting the EMT process, which is a key cellular mechanism in tissue fibrosis. Various types of nanomaterials have been shown to inhibit EMT, suggesting a promising strategy for developing nanomaterials as drug delivery vehicles or cancer-inhibiting agents for effective antimetastatic therapies [49]. EMT is a biological process characterised by the reorganization of cytoskeletal and adhesive structures, leading to reduced cell-cell adhesion and the acquisition of directional motility. In this study, several cell-cell adhesion-related genes, including those associated with fibronectin and collagen, were found to be regulated similarly to fibrosis markers such as Spp1 and Timp1 (Supplementary Figure S10). These findings align with prior research indicating that EMT in alveolar type II (ATII) cells and altered lung fibroblast function are key contributors to CeO_2_NP-induced pulmonary fibrosis [50]. Additionally, previous studies have demonstrated that the TGF-β-mediated Smad signalling pathway plays a pivotal role in the protective effects of CeO_2_NPs [27]. The regulation of EMT via the TGF-β1 signalling pathway has also been linked to the protective effects of other nanomaterials, such as multiwalled carbon nanotubes (MWCNTs) [51] and nickel oxide nanoparticles (NiONPs) [52].

When applying adverse outcome pathways (AOPs) to facilitate mechanistic understanding via an online AOP Wiki resource (https://aopwiki.org/), several AOPs identify lung fibrosis as an adverse outcome (AO). Specifically, AOP241 - Latent TGFβ1 Activation Leading to Pulmonary Fibrosis, characterises lung fibrosis as an AO triggered by nanotubes as a stressor involving the TGFβ signalling pathway [53]. In this context, CeO_2_NPs may act as a molecular initiating event (MIE), activating TGFβ signalling, promoting EMT, and driving collagen accumulation toward the AO of fibrosis. In contrast, under the conditions of the present study, CeO_2_NPs may function as redox-active modulators, suppressing TGFβ activation, inhibiting EMT, and reducing collagen deposition, thereby attenuating fibrotic outcomes during active bleomycin-induced pulmonary injury. These findings suggest that while CeO_2_NPs can act as profibrotic stressors under certain conditions, they may exert protective or modulatory effects within an establishing or established fibrotic microenvironment.

In addition to affecting EMT-related pathways, CeO_2_NPs may impact pulmonary fibrosis by exerting direct effects on key cellular players involved in fibrotic progression, such as fibroblasts and immune cells. CeO_2_NPs can influence fibroblast activation, proliferation, and extracellular matrix production, which are critical steps in the development and maintenance of fibrotic tissue [54]. Additionally, CeO_2_NPs may alter the function and phenotype of immune cells, through impacting their ability to promote or resolve inflammation, and potentially shift macrophage polarization toward profibrotic phenotypes, thereby modulating the inflammatory milieu that drives fibrosis [15].

### In vitro-in vivo comparison

Comparing in vitro studies utilising human cells and in vivo studies using animal models is helpful for advancing new approach methodology (NAM)**-**based approaches. Such comparisons help to improve the predictive accuracy of in vitro models and determine whether they effectively replicate key in vivo mechanisms and disease processes. To date, few studies have directly compared in vivo and in vitro ALI models using the same or similar nanoparticle aerosols. Our previous work with silver nanoparticles (AgNPs) revealed comparable toxicity trends and gene expression changes between nose-only exposed rats and ALI-exposed human SmallAir™ cultures, suggesting that ALI models offer promise for predictive toxicology [36]. In this study, the AE-ALI SmallAir™ model showed comparable LDH release to that of one-week in vivo exposures, suggesting dose-dependent cytotoxic and protective effects of CeO_2_NPs under bleomycin pretreatment, although gene expression responses exhibited partial correlation, likely influenced by inter-donor variability. Careful consideration of dose equivalence and particle characteristics is essential, as differences in agglomeration state, size, and surface area will typically alter the magnitude but not the fundamental mechanisms of toxicity. While the in vitro model used here is suitable for assessing acute epithelial responses in the small airway, its limited cell composition, as it lacks fibroblasts, endothelial cells, and immune cells, constrains its ability to model long-term outcomes such as fibrosis and EMT-driven tissue remodelling. Future studies should incorporate co-culture systems reflective of the range of potentially affected lung regions and both healthy and diseased cells to better mimic the lung’s heterogeneous microenvironment, allowing more predictive assessment of nanoparticle effects across cellular and species contexts. To support this, further comparative studies are needed to guide the appropriate selection of cell models, exposure conditions, and endpoints. Such studies are crucial to ensure that relevant in vivo mechanisms and pathological processes are sufficiently captured by these in vitro systems to ensure meaningful interpretation and to contribute to their validation.

## Conclusions

The aim of this study was to examine how inhaled CeO_2_NPs influence active fibrotic disease processes. In Sprague-Dawley rats, exposure to CeO_2_NPs alone induced inflammatory responses, with fibrotic changes becoming more pronounced over the duration of exposure. As expected, bleomycin treatment increased total BAL cell counts, enhanced fibrotic staining, and upregulated genes associated with inflammation and oxidative stress and these effects were attenuated at fourteen days post-treatment compared with seven days, consistent with partial recovery from bleomycin-induced lung injury. Interestingly, one-week exposure to CeO_2_NPs appeared to modify these effects, reducing fibrotic staining and downregulating genes associated with impaired lung function, inflammation, and EMT. In contrast, two-week exposure to CeO_2_NPs resulted in more severe pulmonary effects compared with the one-week exposure, making it difficult to discern whether CeO_2_NPs interfered with active bleomycin-driven fibrotic processes or predominantly reflected nanoparticle-induced toxicity. These findings indicate that the timing of CeO_2_NP exposure relative to disease progression is critical for detecting their modulatory effects in the bleomycin-induced disease models, and that such interactions are most informative during the active disease development phase rather than during the recovery phase following bleomycin-induced injury. To further explore these responses at the cellular level, 3D human small airway epithelium cultures (SmallAir™) were exposed to CeO_2_NP aerosols at the air-liquid-interface. The in vitro results indicated that certain cellular responses induced by bleomycin could be mitigated by CeO_2_NP aerosol exposure. However, the variable regulation of gene expression highlights the limitations of the cellular model approach in fully capturing the complexity of in vivo disease processes. Overall, the results of the present study suggest that inhaled CeO_2_NPs may reduce the severity of some inflammatory or fibrotic diseases through impacting disease progression pathways. These findings imply that hazard assessment of CeO_2_NPs should carefully consider both their potential adverse and protective effects, particularly in the context of pre-existing disease or tissue injury, as their effects may not follow the conventional paradigms of nanoparticle toxicity.

## Supplementary Information

Below is the link to the electronic supplementary material.

Supplementary Material 1.

## Data Availability

Sequence data that support the findings of this study are available upon request.

## Abbreviations

NM: Nanomaterials
CeO_2_NP: Cerium(IV) oxide nanoparticle
PM: Particulate matter
COPD: Chronic obstructive pulmonary disease
HDM: House dust mite
TEM: Transmission electron microscope
DLS: Dynamic light scattering
SD: Sprague-Dawley
PBS: Phosphate buffered saline
i.t.: Intratracheal instillation
IPF: Idiopathic pulmonary fibrosis
Bleo: Bleomycin
BALF: Bronchoalveolar lavage fluid
H&E: Haematoxylin and eosin
MT: Masson’s trichrome
PCA: Principal component analysis
RPKM: Reads per kilobase per million mapped reads
GSEA: Gene set enrichment analysis
IPA: Ingenuity pathway analysis
MPPD: Multiple-path particle dosimetry
DE: Deposition efficiency
ALI: Air-liquid interface
AE-ALI: Aerosol exposure at the air-liquid-interface
ICP-MS: Inductively coupled plasma-mass spectrometry
IC: Incubator control
SC: System control
LD: Low dose
HD: High dose
LDH: Lactate dehydrogenase
SD: Standard deviation
PP: Primary particle
CMD: Count median diameter
PDI: Polydispersity index
GSD: Geometric standard deviation
DEG: Differential expressed gene
FDR: False discovery rate
EMT: Epithelial-mesenchymal transition
BAL: Bronchoalveolar lavage
ROS: Reactive oxygen species
MWCNT: Multiwalled carbon nanotube
NiONP: Nickel oxide nanoparticle
AOP: Adverse outcome pathway
AO: Adverse outcome
MIE: Molecular initiating event
AgNP: Silver nanoparticle
