# Supplementary material for: Modulatory effects of CeO2 nanoparticles on bleomycin-induced active pulmonary disease processes in animal and human airway epithelium models
Source: Part Fibre Toxicol. 2026 Jan 16;23:5. doi: 10.1186/s12989-026-00658-9 (PMC12829198; doi:10.1186/s12989-026-00658-9)
Supplement: Supplementary file 1 — Supplementary Material 1. [file 12989_2026_658_MOESM1_ESM.docx]

**Supplementary information**

**Modulatory effects of CeO₂ nanoparticles on bleomycin-induced active pulmonary disease processes in animal and human airway epithelium models**

Chang Guo ^a, b^, Alison Buckley ^a, b^, Sarah Robertson ^c^, Adam Laycock ^a, b^, Xianjin Cui ^d^, Eugenia Valsami-Jones ^d^, Tim Gant ^a, b^, Martin O. Leonard ^a, b^, Rachel Smith ^a, b^

^a^ Toxicology Department, Radiation, Chemicals, Climate and Environmental Hazards Directorate (RCE), UK Health Security Agency (UKHSA), Harwell Campus, Oxfordshire OX11 0RQ, UK

^b^ The National Institute for Health Research Health Protection Research Unit (NIHR HPRU) in Environmental Exposures and Health (EEH) at Imperial College London in partnership with UKHSA

^c^ Public Health Scotland (PHS), Gyle Square, 1 South Gyle Crescent, Edinburgh, EH12 9EB

^d^ School of Geography, Earth and Environmental Sciences, University of Birmingham, Birmingham B15 2TT, UK

**Methods**

Cerium Dioxide Nanoparticles

*Supplementary Section 1. Synthesis and Purification Method*

CeO_2_NPs for both in vivo and in vitro exposure were provided by the University of Birmingham. The method for CeO_2_NP synthesis and purification has been described previously [1]. In brief, a cerium stock solution was prepared by dissolving 21.7 g of cerium nitrate hexahydrate in 100 mL deionized (DI) water, and a sodium hydroxide stock solution was prepared by dissolving 2 g of NaOH in 50 mL of DI water. Then, 8.5 mL of Ce stock solution, 5 mL of NaOH stock solution and 70 mL of DI water were mixed by magnetic stirring for 1 h followed by heating at 150 ◦C in an autoclave for 24 h. The prepared product was washed four times with DI water via centrifugation. The solids collected in the last centrifugation were redispersed in DI water. The mixture was placed in a bath of sodium chloride and ice for 3 h. After centrifugation, the white solids were removed leaving a transparent solution which was then filtered through a Millipore system to remove larger agglomerates. The solution was then dried via a vacuum pump for several hours at room temperature to produce a light-yellow powder. To create the CeO_2_NP stock suspensions, the particles were resuspended in DI water and dispersed via an ultrasonic bath. The suspension concentration used to achieve the required dose in the in vitro exposures (3.4 mg/mL) was ~3 times greater than that required for the in vivo exposures (1.0 mg/mL).

*Supplementary Section 2. Comparison of in vivo and in vitro CeO_2_NP stock suspensions*


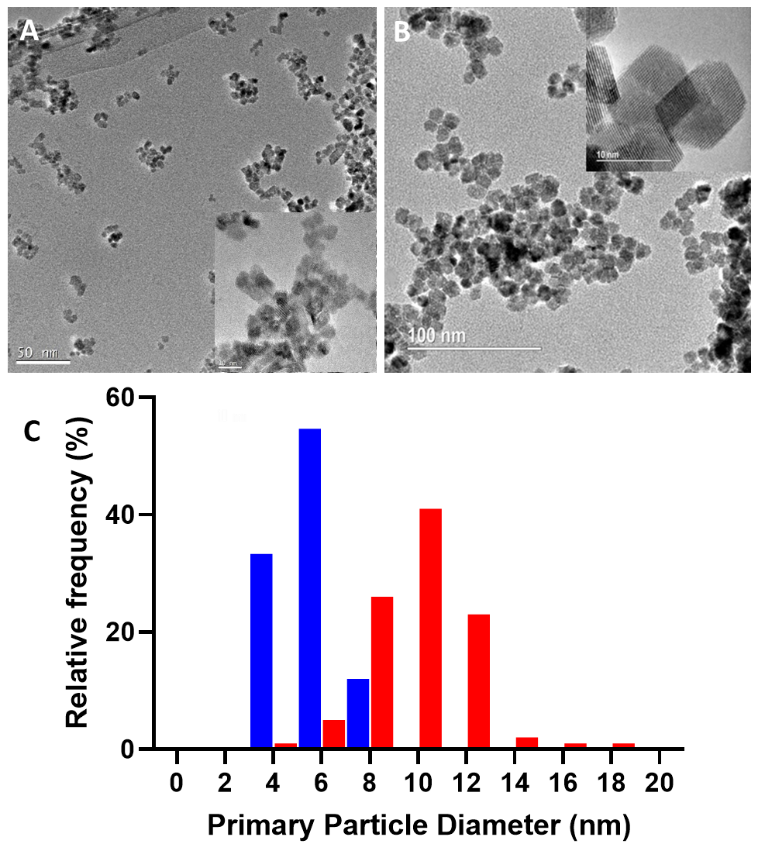


**Supplementary Figure S1.** CeO_2_NP stock suspension primary particles. Representative TEM images of the particles in suspension for A) the in vivo stock suspension (1 mg/mL) and B) the in vitro stock suspension (3.4 mg/mL). C) shows the frequency distribution of the primary particle size measured from the TEM images with the in vivo results (N=75) in blue and the in vitro results in red (N=100). For the in vivo stock suspension, the size distribution was determined from 75 particles, randomly selected from 5 TEM images taken at either 250000X or 500000X magnification and for the in vitro stock suspension, the size distribution was determined from 100 particles randomly selected from 13 TEM images taken at a range of magnifications between 120000X and 2000000X via the image processing software ImageJ [2]. The diameter here is defined as the average length of two tangents (approximately at right angles to each other) drawn across the primary particle. The average (± standard deviation) primary particle diameter was 5.6 ± 1.1 nm for the in vivo stock and 9.9 ± 1.9 nm for the in vitro stock.

Experimental design of in vivo animal exposure studies

*Supplementary Section 3. In vivo exposure system and aerosol characterization*

The nose-only inhalation exposure system and the aerosol characteristics used are described in detail in Guo et al [3]. In brief, the CeO_2_NP stock suspension was aerosolised into filtered, compressed air using two constant output atomisers in parallel (model 3076, TSI Inc., Shoreview, MN, USA,) dried, charge neutralised and then passed to a custom-built nose-only exposure manifold (EMMS, Bordon, UK) [4]. The aerosol mass concentration (TEOM model 1400a, Thermo Scientific, Franklin MA, USA), number concentration (CPC model 3775, TSI Inc., Shoreview, MN, USA) and size distribution (SMPS model 3936N76, TSI Inc., Shoreview, MN, USA) were monitored in real-time throughout the exposure period, the average mass concentration for dose estimation was determined gravimetrically and the aerosol particles were captured onto TEM grids (lacey carbon film 400 mesh copper grids, S166-4, Agar Scientific Ltd., Standsted, Essex, UK) for high resolution TEM (JEOL 3000F, JEOL Inc. Tokyo, Japan) via a Mini Particle Sampler (Ecomesure, Saclay, France). The aerosol temperature, relative humidity and oxygen content were monitored and maintained throughout exposures for animal comfort. For the control exposures, Milli-Q water was used to replace the CeO_2_NP suspension.

In vitro/in vivo dose matching

*Supplementary Section 4: In vivo dose estimation*

Estimates of the deposited dose, D (μg), in the lung and alveolar regions were determined using the following formula, D = C × MV × T × DE × 10^-3^, where C (mg/m^3^) is the aerosol mass concentration, MV (mL/min), the rat minute ventilation, T (min), the exposure duration, and DE, the deposition efficiency. The minute ventilation was measured using head-out plethysmographs (EMMS, Bordon, UK) for 4 rats during 3-hour exposures on different days, as detailed in Guo et al [3]. The average MV was 209 mL/min, which is in line with typical values for animals of similar mass found in the literature [5-8]. The deposition efficiencies for the different regions of the respiratory tract were determined using the multiple-path particle dosimetry (MPPD) model (version 2.11, Applied Research Associates, Inc.) [9]. Using the average measured breathing frequency, tidal volume and minute ventilation, deposition efficiencies were calculated for the minimum (250 g) and maximum (320 g) rat body weight. The average was then used to calculate dose. Table S1 below outlines the MPPD model parameters used for the minimum and maximum body weights and the minimum, maximum and average deposition efficiencies calculated. The term “small airways” is used here to refer to generations 9-15. In practise, the deposition efficiencies estimated for this region, and therefore the dose estimates, are not very different to those calculated for MPPD’s default tracheobronchial (TB) region, bearing in mind the uncertainties in the modelling process. Both are given in the tables for comparison.

| **MPPD Input Parameters** | | **Lower bodyweight** | **Upper bodyweight** | **Average** |
| --- | --- | --- | --- | --- |
| Airway Morphometry | Species | Rat | Rat |  |
|  | Model | Asymmetric SD | Asymmetric SD |  |
|  | Body Weight (g) | 250 | 320 |  |
|  | FRC (mL) | 3.15404 | 3.6127 |  |
|  | URT volume (mL) | 0.36452 | 0.42882 |  |
| Particle Properties | Density (g/cm^3^) | 4.4 | 4.4 |  |
|  | Aspect Ratio | 1 | 1 |  |
|  | Diameter, CMD (μm) | 0.044 | 0.044 |  |
|  | GSD (diam.) | 1.7 | 1.7 |  |
|  | GSD (length) | 1 | 1 |  |
|  | Correlation | 0 | 0 |  |
|  | Single/Multiple/multimodel | Single | Single |  |
| Constant exposure conditions | Body orientation | Stomach | Stomach |  |
|  | Aerosol conc (mg/m^3^) | 1.8 | 1.8 |  |
|  | Breathing freq (/min) | 152 | 152 |  |
|  | tidal volume (mL) | 1.375 | 1.375 |  |
|  | inspiration fraction | 0.5 | 0.5 |  |
|  | pause fraction | 0 | 0 |  |
|  | breathing scenario | nose-only | nose-only |  |
| **Calculated deposition efficiencies** | Head | 0.0856 | 0.0875 | 0.08655 |
|  | TB | 0.0573 | 0.0523 | 0.0548 |
|  | Pulmonary | 0.1568 | 0.1224 | 0.1396 |
|  | Total | 0.2998 | 0.2623 | 0.28105 |
|  | Small Airways (Gen 9-15) | 0.0486 | 0.0441 | 0.04635 |

**Supplementary Table S1.** MPPD parameters used to estimate deposition efficiencies in the different regions of the lungs for the in vivo exposures. Deposition efficiencies were calculated for the minimum (250 g) and maximum (320 g) rat body weight, and then averaged.

The exposure conditions and lung region surface areas used to calculate dose and dose per unit area, alongside the calculated doses for each region are given in Table S2 below.

|  | | **Lower bodyweight** | **Upper bodyweight** | **Average** |
| --- | --- | --- | --- | --- |
| Surface areas | TB (cm^2^) | 22.5 | 22.5 |  |
|  | Pulmonary (cm^2^) | 3400 | 3400 |  |
|  | Small Airways (Gen 9-15) | 19.137 | 20.952 |  |
| Exposure conditions | Low dose duration (mins) | 720 | 720 |  |
|  | High dose duration (mins) | 1440 | 1440 |  |
|  | MV (measured) (mL/min) | 209 | 209 |  |
|  |  |  |  |  |
| **Doses (µg)** | Small airways LD | 13 | 12 | 13 |
|  | Small airways HD | 26 | 24 | 25 |
|  |  |  |  |  |
|  | Alveolar LD | 42 | 33 | 38 |
|  | Alveolar HD | 85 | 66 | 76 |
|  |  |  |  |  |
|  | TB LD | 16 | 14 | 15 |
|  | TB HD | 31 | 28 | 30 |
| **Dose per unit area (ng/cm^2^)** | Small airways LD | 688 | 570 | 629 |
|  | Small airways HD | 1376 | 1140 | 1258 |
|  |  |  |  |  |
|  | Alveolar LD | 12 | 10 | 11 |
|  | Alveolar HD | 25 | 20 | 22 |
|  |  |  |  |  |
|  | TB LD | 690 | 630 | 660 |
|  | TB HD | 1380 | 1259 | 1319 |

**Supplementary Table S2.** Lung region surface areas and exposure conditions used, in addition to the information given in Table S1 to calculate dose and dose per unit area for the low (LD) and high (HD) dose in vivo exposures.

*Supplementary Section 5. Preliminary Investigation to Determine In vitro Deposition Efficiency*

To determine the deposition efficiency of the in vitro exposure system a series of preliminary sham exposures were carried out. The deposited aerosols were collected via dummy, stainless steel cell culture inserts with cellulose nitrate (CN) membrane filters (WCN, 0.8 μm pore size; Cytiva Whatma^TM^, Marlborough, MA, USA) clamped at the bottom. The mass deposited in all 3 wells for each exposure run was determined via ICP-MS (details on the method described below). Seven, 30 min exposures were carried out using the CeO_2_NP stock suspension at aerosol concentrations ranging from 3 x 10^5^ to 5.5 x 10^6^ particles.cm^-3^ (0.8-43 mg.m^3^).

The deposition efficiency (*DE*) was then calculated as the ratio of the deposited mass, *M_CeO2_* (ng), to the mass delivered to the insert via the following equation

$DE=1000.\frac{M_{CeO2}}{Q.t.C_{M,ae}}$

where *Q* (mL/min) is the aerosol flow rate delivered to each well, *t* (min) is the exposure duration and *C_M,ae_* (µg/m^3^) is the average aerosol mass concentration.

The DE results as a function of aerosol concentration are shown in the figure below. Excluding the lowest aerosol concentration where the average DE > 100% suggested inaccuracy in the deposited or aerosol mass calculations, the average (± standard deviation) deposition efficiency was 39 ± 10%, with a significant difference (p < 0.001) found between the exposure sets (one-way ANOVA) and a slight, negative linear trend (p < 0.01) found between the DE and aerosol concentration. No trends in consistently high or low doses were observed across the 3 wells.

**Supplementary Figure S2**. Deposition efficiency as a function of aerosol number concentration. Each point represents the average of 3 wells for a single 30 min exposure, with the error bars indicating the standard deviation.

In vitro AE-ALI exposure system and aerosol characterization

*Supplementary Section 6. Dose determination via ICP-MS*

The deposited dose of CeO_2_ was measured via CN filter membranes held at the bottom of stainless-steel “dummy” cell culture inserts in one or more of the exposure wells. It has previously been shown that for this system and CeO_2_NP aerosols, the mass deposited onto CN filters is equivalent to that deposited onto cells [10], although this may not be the case for all combinations of systems, filter membranes and aerosols. The mass of cerium (Ce-140) deposited can then be determined via inductively coupled plasma mass spectrometry (ICP-MS). The complete filter samples were transferred to microwave digestion vessels where 1.5 mL of HNO_3_, 1 mL of ultrapure water and 0.5 mL of H_2_O_2_ were added before processing in an Anton Paar Multiwave Go Plus microwave digester by ramping to 180 °C over 10 min and holding for additional 20 min. The sample digests were further diluted with ultrapure water and analysed via a Thermo Scientific iCAP Q ICP-MS instrument calibrated with mixed element standards to quantify the total mass of Ce on the sample, from which the mass of CeO_2_ was calculated stoichiometrically.

*Supplementary Section 7. Spatial distribution visualisation via laser ablation ICP-MS*

The spatial distribution of cerium (Ce-140) on the CN filter membranes or bottom of the Transwell cell culture inserts was determined via laser ablation ICP-MS via a New Wave Research NWR213 laser ablation system (Elemental Scientific Instruments, Omaha, Nebraska, USA) coupled to an iCAP Q ICPMS (Thermo Scientific, Hemel Hempstead, UK). A laser spot size of 100 μm in diameter, fluence of 3 J cm^-2^, scan speed of 182 μ/ms and repetition rate of 20 Hz were used. The cell gas was helium, which was run at a flow rate of 0.8 mL/min. Helium was also used as the collision gas in KED mode for both the solution and laser ablation ICP-MS. Image generation was achieved via Iolite v3 [11] within Igor Pro 6.36 (Wavemetrics Inc. Oregon, USA)

*Supplementary Section 8. In vitro experiment using AE-ALI to determine the optimal conditions for bleomycin treatment*

A pilot experiment was conducted using the same cellular model to establish conditions for bleomycin treatment (**Supplementary Figure S3**). General toxicity was assessed by measuring lactate dehydrogenase (LDH) levels in the culture medium, whereas oxidative stress and inflammatory responses were evaluated through gene expression analysis of small airway-specific markers (e.g., MUC5AC and SLC26a4) and genes responsive to oxidative stress and inflammation (e.g., HMOX1, LCN2, CXCL1, and IL-8). A bleomycin concentration of 50 µg/mL, which led to a significant increase in general toxicity and oxidative stress, was selected for subsequent exposure to CeO_2_ aerosol particles.


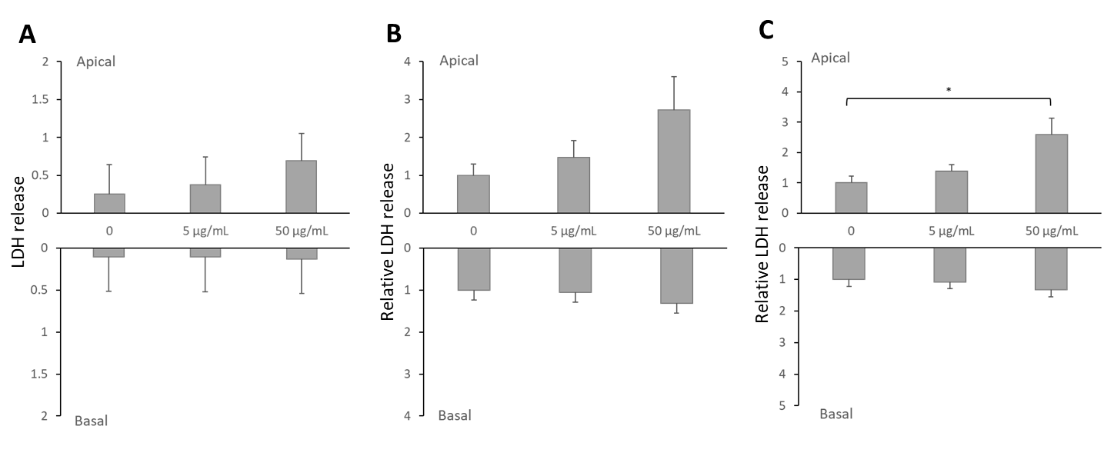


**Supplementary Figure S3** Cytotoxicity analysis of SmallAir^TM^ at 1 day following exposure to bleomycin alone. (A) LDH release was measured in both the apical and basal media. (B) Relative LDH release in the apical and basal media was normalized to the average of LDH release in the 0 µg/mL bleomycin group. (C) Relative LDH release in the apical and basal media was normalized to that of the individual donor control (bleomycin 0 µg/mL group). The data are shown as the means ± SDs. Compared with that in the 0 µg/mL bleomycin group, the difference was statistically significant (* p < 0.05).


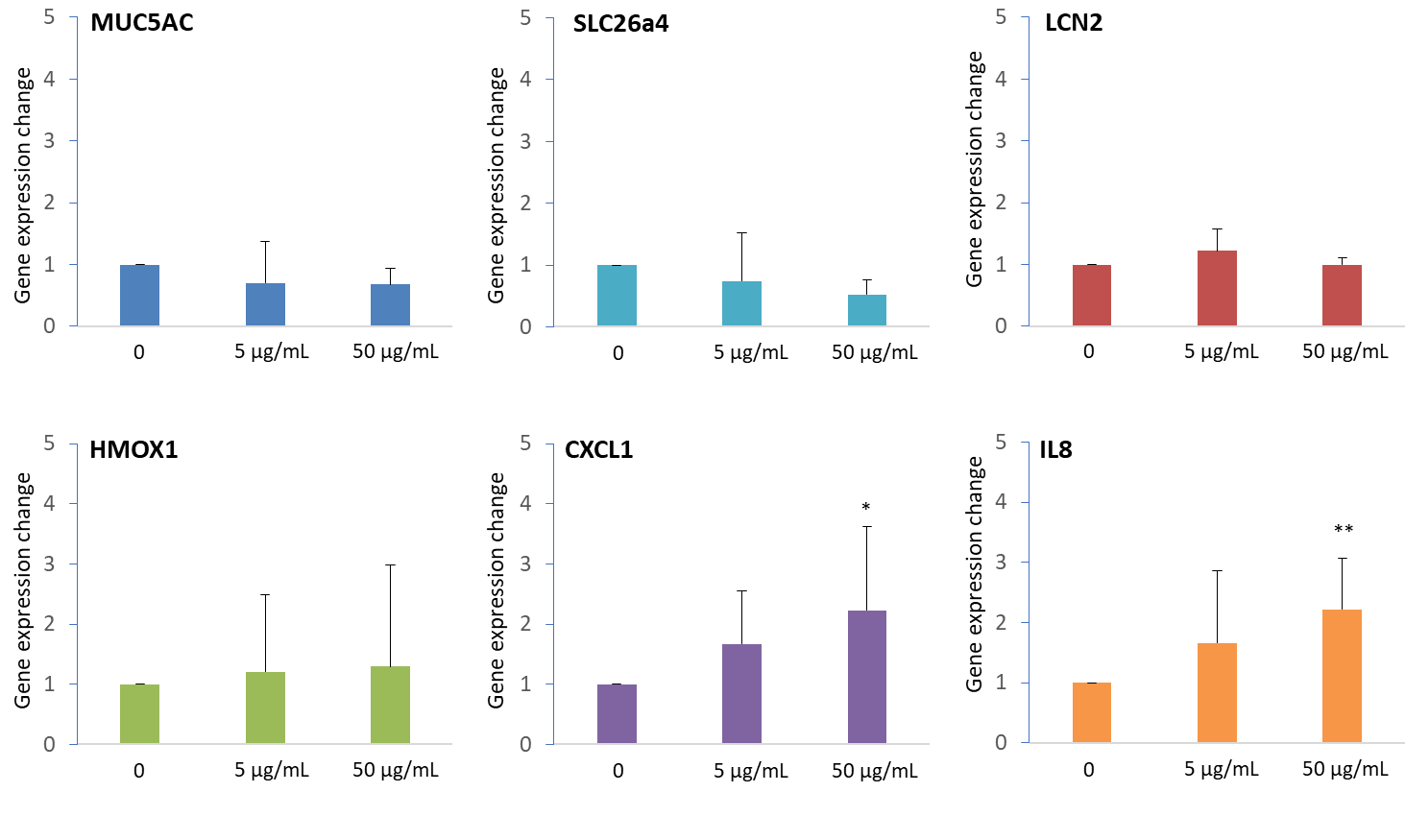


**Supplementary Figure S4** Expression of selected genes in SmallAir^TM^ at at 1 day following exposure to varying concentrations of bleomycin (0, 5, and 50 µg/mL). Gene expression alterations were normalized to those of the respective controls from individual donors. The data are shown as the means ± SDs. Statistical significance between the exposed groups (bleomycin at 5 and 50 µg/mL) and the control group (bleomycin at 0 µg/mL) was assessed via paired t tests (* p < 0.05, ** p < 0.01).

*Supplementary Section 9. Pathway analysis*

The RNA sequencing data were processed for pathway analysis via Ingenuity Pathway Analysis (IPA) software (Qiagen, UK). The data were pre-processed to eliminate potential outliers via hierarchical clustering via Qlucore Omics Explorer software (Qlucore, Sweden). Those samples that did not cluster with the treatment groups were excluded from subsequent analysis. Differential gene expression (DEG) data were generated as input metrics for pathway analysis via Qlucore software (nominal p value <0.005). Canonical pathways were chosen within IPA to capture the cellular signalling pathways of interest. Pathways significantly associated with the input RNA sequencing DEGs were ranked by p-value.

*Supplementary Section 10. Primers used in the PCR analysis*

**Supplementary Table S3.** Forward and reverse sequences of the primers used in the present study

| Gene | Forward sequence (5’-3’) | Reverse sequence (5’-3’) |
| --- | --- | --- |
| HPRT1 | TCAGGCAGTATAATCCAAAGATGGT | AGTCTGGCTTATATCCAACACTTCG |
| HMOX1 | TCACTGTGTCCCTCTCTC | ATGGTCCTGGATGTGCTTT |
| LCN2 | AGACAAAGACCCGCAAAAG | TGGCAACCTGGAACAAAAG |
| CXCL1 | AACCGAAGTCATAGCCACAC | GTTGGATTTGTCCTGTTCAGC |
| IL-8 | TTGGCAGCCTTCCTGATTTC | AACTTCTCCACAACCCTCTG |
| MUC5AC | GGAACTGTGGGGACAGCTCTT | GTCACATTCCTCAGCGAGGTC |
| TGFB3 | CTGGATTGTGGTTCCATGCA3 | TCCCCGAATGCCTCACAT |
| FN1 | GCTCAGCAAATGGTTCAG | GTCTCTTCAGCTTCAGGTTTA |
| SLC7A11 | GGGCATGTCTCTGACCATCT | GTTCCACCCAGACTCGTACA |
| TXNRD1 | TTTCCGTGCCCAAATCCAAG | CCTGCCAAATGTCAGCTTCA |

**Results**

In vivo and in vitro doses

*Supplementary Section 11. Visualisation of in vitro dosing*

The spatial distribution of CeO_2_NPs deposited onto the cells during in vitro exposure was visualised via laser ablation ICP-MS and TEM. While laser ablation ICP-MS shows the distribution across the entire area of the cell culture insert, TEM shows greater detail over smaller areas as well as the form in which the particles are present. The laser ablation images, an example of which is shown in Figure 3, show that the distributed of Ce-140 (assumed to be evenly distribution throughout the CeO_2_NPs) is uniform across the majority of the base of the cell culture insert, with only a slight decrease in concentration around the edges. Both the low- and high dose treatment resulted in similar patterns of particle distribution. It has previously been shown that the distribution is equivalent across the 3 exposure wells in the Cultex RFS [10]. The TEM images (Figure 4) show that the shape and size of the CeO_2_NP agglomerates deposited are the same as those observed in the aerosol samples (Figure 2) and that they are distributed evenly across the area sampled by the TEM grid (which was placed in the centre of the cell culture insert). As expected, under high-dose exposure, a greater concentration of particles were deposited than under low-dose exposure, and the shape and size of the agglomerates, however remained unchanged.

**
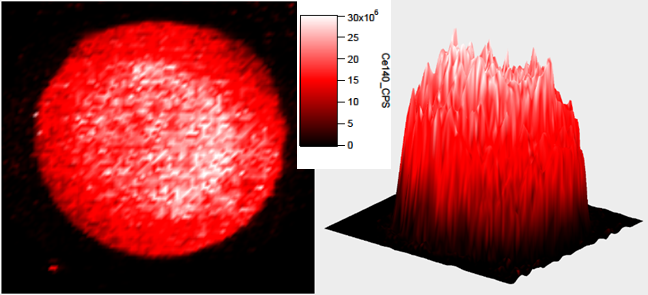
**

**Supplementary Figure S5.** 2D (left) and 3D (right) representations of the spatial distribution of Ce-140 across the bottom of the cell culture insert during a high dose in vitro exposure, as measured via laser ablation ICP-MS. The concentration scale is in counts per second.

***
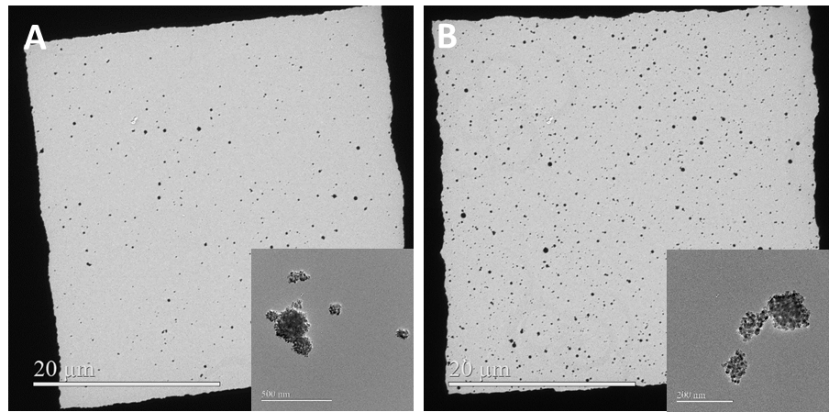
***

**Supplementary Figure S6*.*** Example TEM images of a TEM grid placed in the centre on the bottom of a dummy cell culture insert during (A) low- and (B) high-dose exposure.

*Supplementary Section 12. Biological endpoint analysis of the in vivo exposure study*


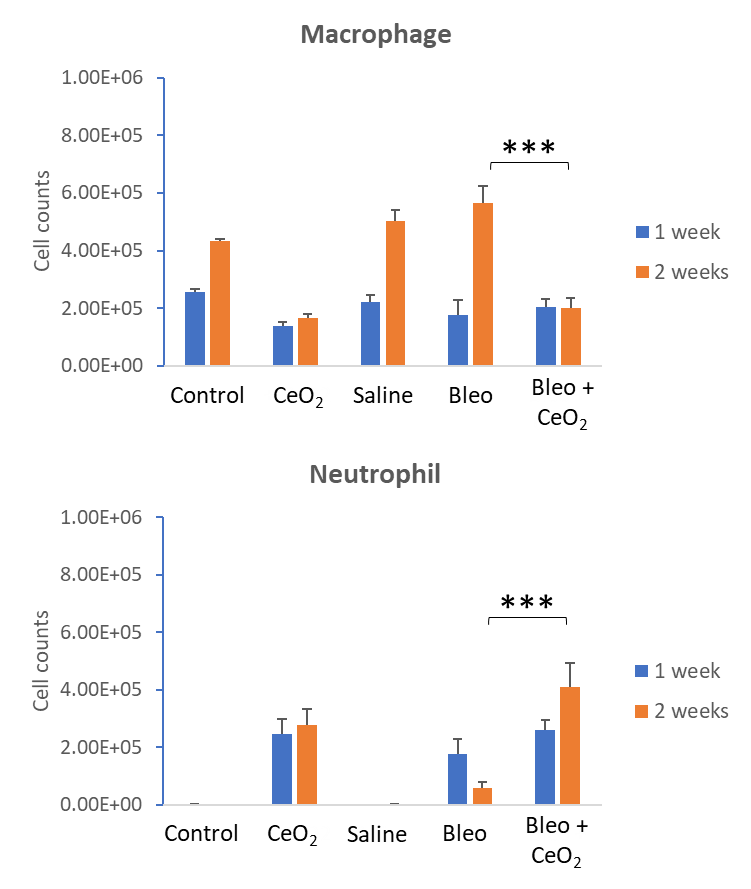


**Supplementary Figure S7** Absolute numbers of macrophages and neutrophils after the inhalation of CeO_2_NPs or control aerosols. The data are presented as the means ± SDs (n = 5 rats per group for exposed and n = 3 for controls). * P < 0.05, ** P < 0.01, *** P < 0.001.


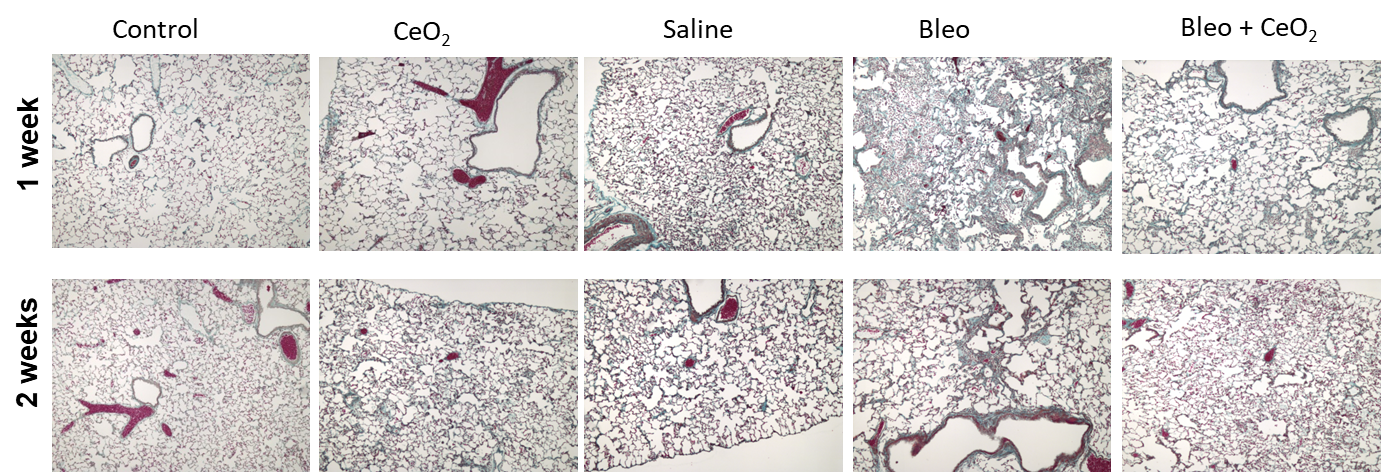


**Supplementary Figure S8** Representative Masson’s Trichrome-stained lung sections from rats in different control and exposure conditions.


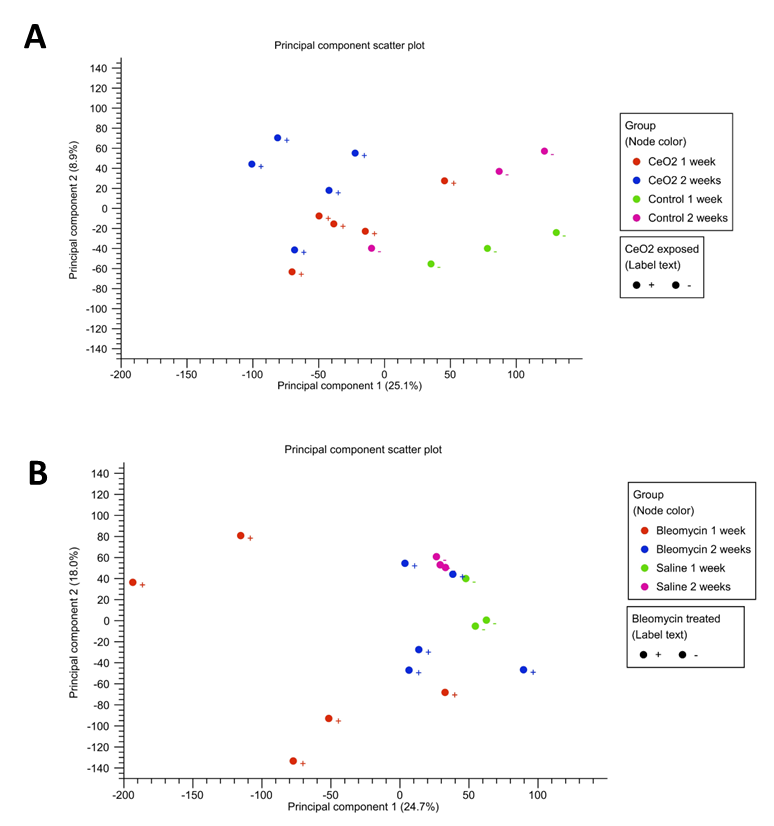


**Supplementary Figure S9** Principal component plots. (A) Groups exposed to CeO_2_NPs or control aerosols for 1 week or 2 weeks. (B) Bleomycin pre-treatment groups.


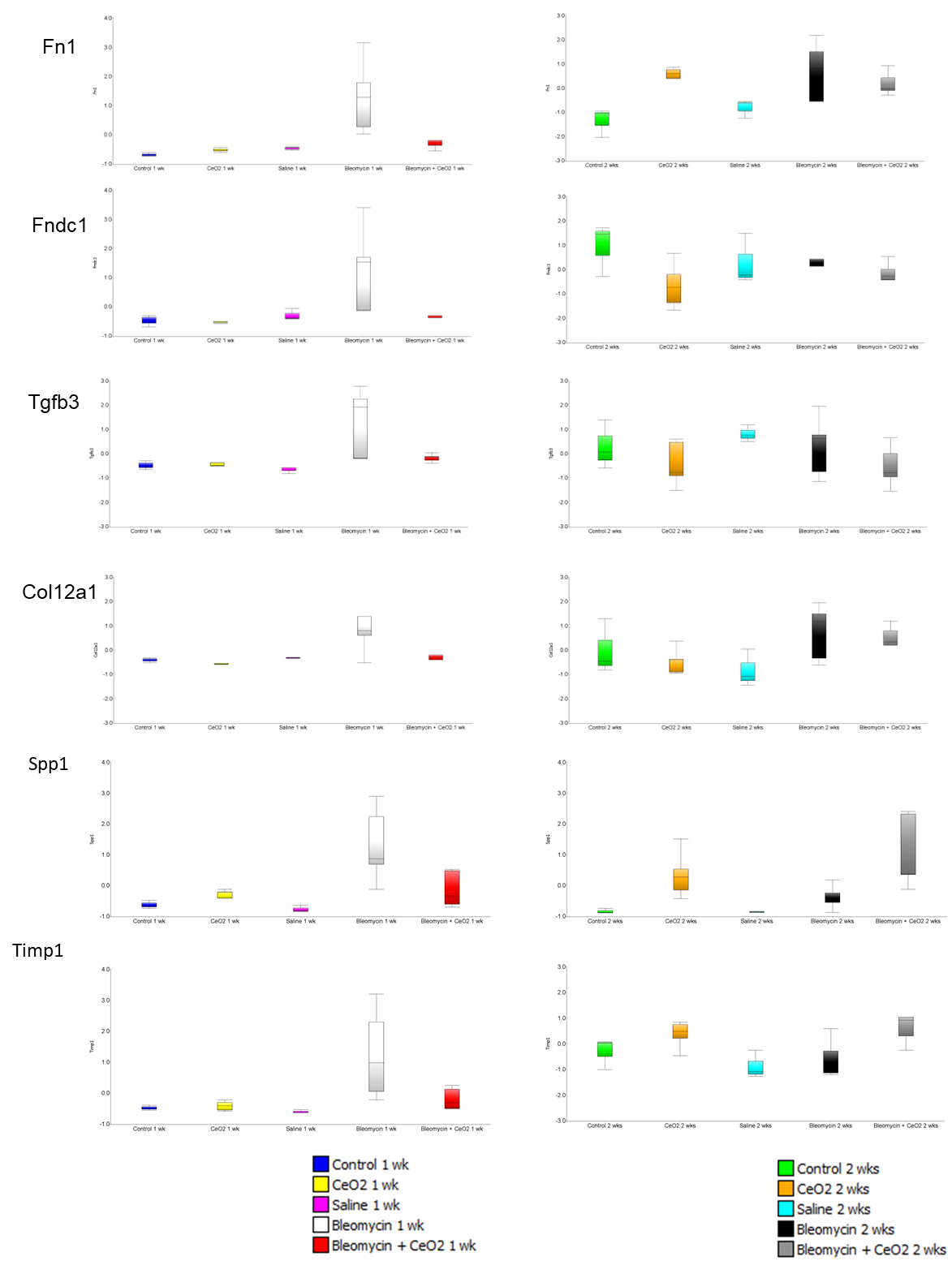


**Supplementary Figure S10** Expression of selected genes related to epithelial-mesenchymal transition (EMT) in the lungs following various exposures.

*Supplementary Section 13. Alternative pathway analysis via IPA*

Examination of transcriptomic responses to bleomycin alone revealed 248 differentially regulated genes (DEGs)1 week after bleomycin exposure (**Supplementary Figure S11A**). This number was reduced to 9 genes at 2 weeks after the single bolus of bleomycin, indicating a diminished active cellular response to this toxic insult. Ingenuity pathway analysis of the 1-week DEG, revealed the most significantly upregulated pathways as those involved in DNA damage (highlighted in red in **Supplementary Figure S11B**). This response is consistent with the molecular mechanism of bleomycin toxicity within the lung (PMID: 18492718)

Analysis of the effects of CeO_2_NP aerosol exposure for 4 days beginning 24 hrs after a single bolus of bleomycin was carried out at 3 days after the final CeO_2_NP aerosol exposure (**Supplementary Figure S11C**). This resulted in 314 genes differentially regulated in the “Bleo + CeO_2_” group compared with the control (saline + air exposed) groups. These genes appeared to constitute the same cohort of genes originally regulated by bleomycin alone at the same time point. To investigate this further, we selected whose expression was induced by bleomycin alone with > 2-fold change and then further altered by CeO_2_NP aerosols in the opposite direction. This resulted in 221 genes. Pathway analysis of this cohort revealed that DNA damage pathways (highlighted in red in **Supplementary Figure S11D**) were the pathways most differentially regulated. These data suggest that CeO_2_NP aerosol exposure prevents the DNA damage signalling gene response induced by bleomycin.

Analysis of the effects of CeO2NP aerosol exposure on 8 non-consecutive (2 sets of 4 days of exposure across 2 weeks) beginning 24 hrs after a single bolus of bleomycin was carried out at 3 days after the final CeO_2_NP aerosol exposure (**Supplementary Figure S11E**). This resulted in 283 genes differentially regulated in the “Bleo + CeO_2_” group compared with the control (saline + air exposed) group. These genes did not overlap with bleomycin alone at the same time point. Bleomycin alone did not induce significant alterations in gene expression 2 weeks after single bolus exposure. Pathway analysis of the 283 genes revealed that immune cell and inflammatory pathway activation (highlighted in green in **Supplementary Figure S11F**) was the most differentially regulated pathway. These genes were very similar to those genes whose expression was altered by CeO_2_NP aerosol exposure alone at 2 weeks. The interpretation of thses data indicates that CeO_2_NP aerosol exposure is the dominant adverse factor at this time point.


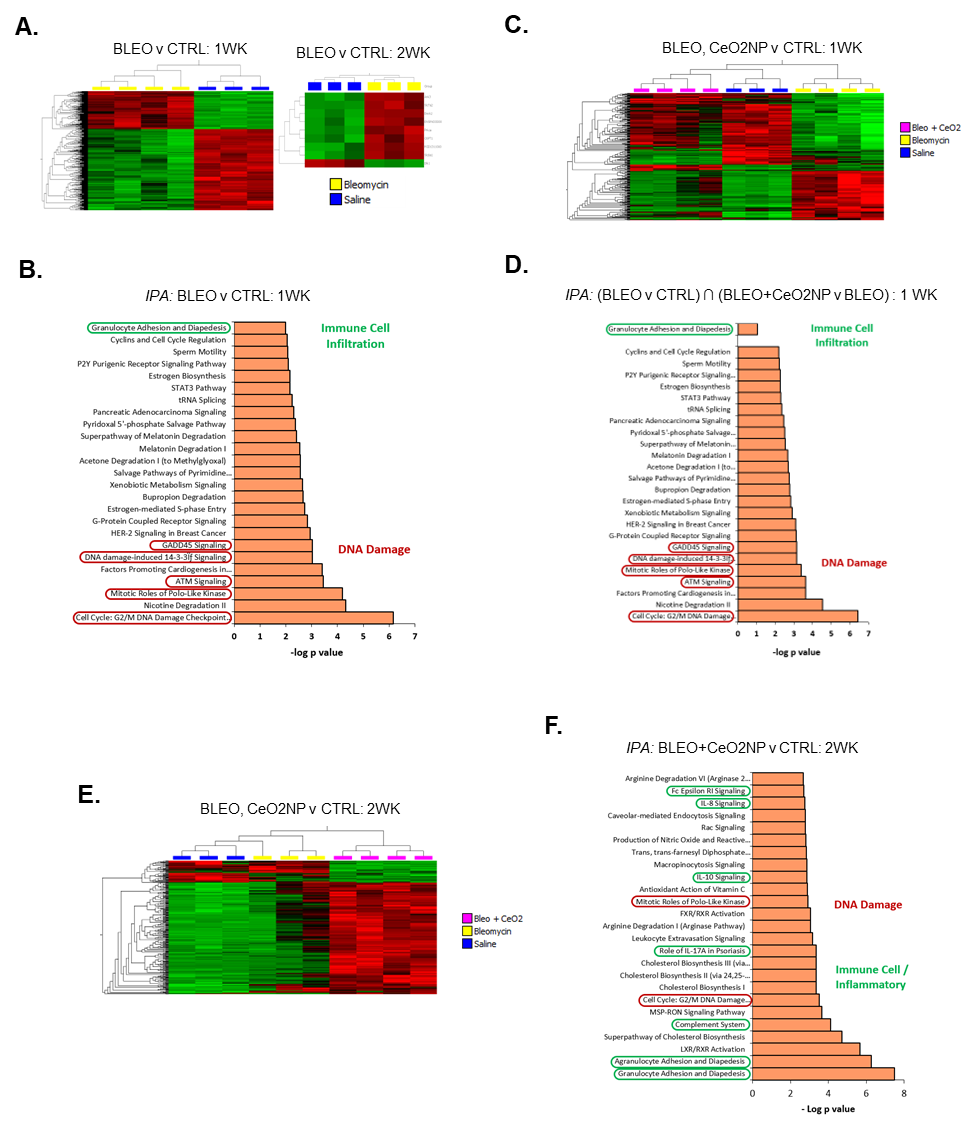


**Supplementary Figure S11**. Pathway analysis of differentially expressed genes (DEGs) in bleomycin-treated and CeO_2_NP-exposed lung tissue. Lung tissue was analyzed for gene expression via RNA sequencing analysis after treatment with bleomycin and CeO_2_NP. Those genes with a nominal p value < 0.005 for treatment versus control samples were selected for further analysis. Hierarchical clustering was used to eliminate outlier samples, and the resulting data were visualized as a heatmap (expression normalized for each gene individually the and normalized to a maximum of 1), where red represents high expression and green represents low expression of genes (A, C, E). Pathway analysis was carried out on selected gene lists as indicated in (B, D, E). DNA damage and immune cell/inflammatory-associated pathways are highlighted.

*Supplementary Section 14. Adverse effects of nanosized CeO2NP aerosols at the ALI prior to bleomycin treatment*

**
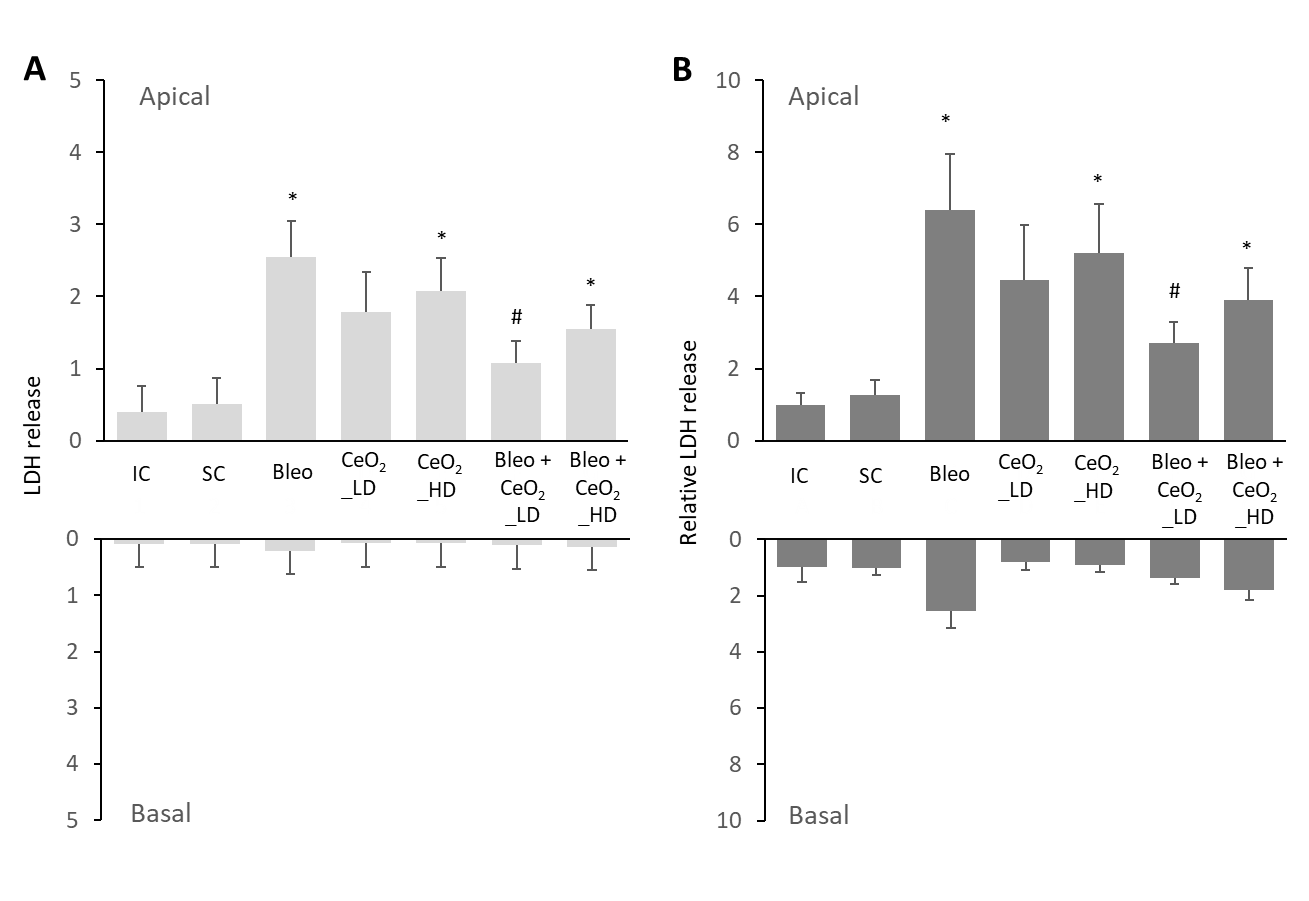
**

**Supplementary Figure S12** Cytotoxicity analysis of SmallAir^TM^ at 1 day following various exposure conditions (IC: incubator control; SC: system control – dried H_2_O aerosol exposure; Bleo: bleomycin + dried H_2_O aerosol exposure; CeO_2_NP_LD: CeO_2_NP aerosol exposure at a low dose of 275 ± 77 ng/cm^2^; CeO_2_NP_HD: CeO_2_NP aerosol exposure at a high dose of 706 ± 151 ng/cm^2^; Bleo + CeO_2_NP_LD: bleomycin + CeO_2_NP aerosol exposure at the low dose; Bleo + CeO_2_NP_HD: bleomycin + CeO_2_NP aerosol exposure at the high dose. (A) LDH release as measured in both the apical and basal media. (B) Relative LDH release in the apical and basal media as normalized to the average of LDH release in the incubator control group. The data are shown as the means ± SDs. Compared with the system control condition, the difference was statistically significant (* p < 0.05). Compared with the condition of Bleo (bleomycin + dried H_2_O aerosol exposure) condition, the difference was statistically significant (# p < 0.05).


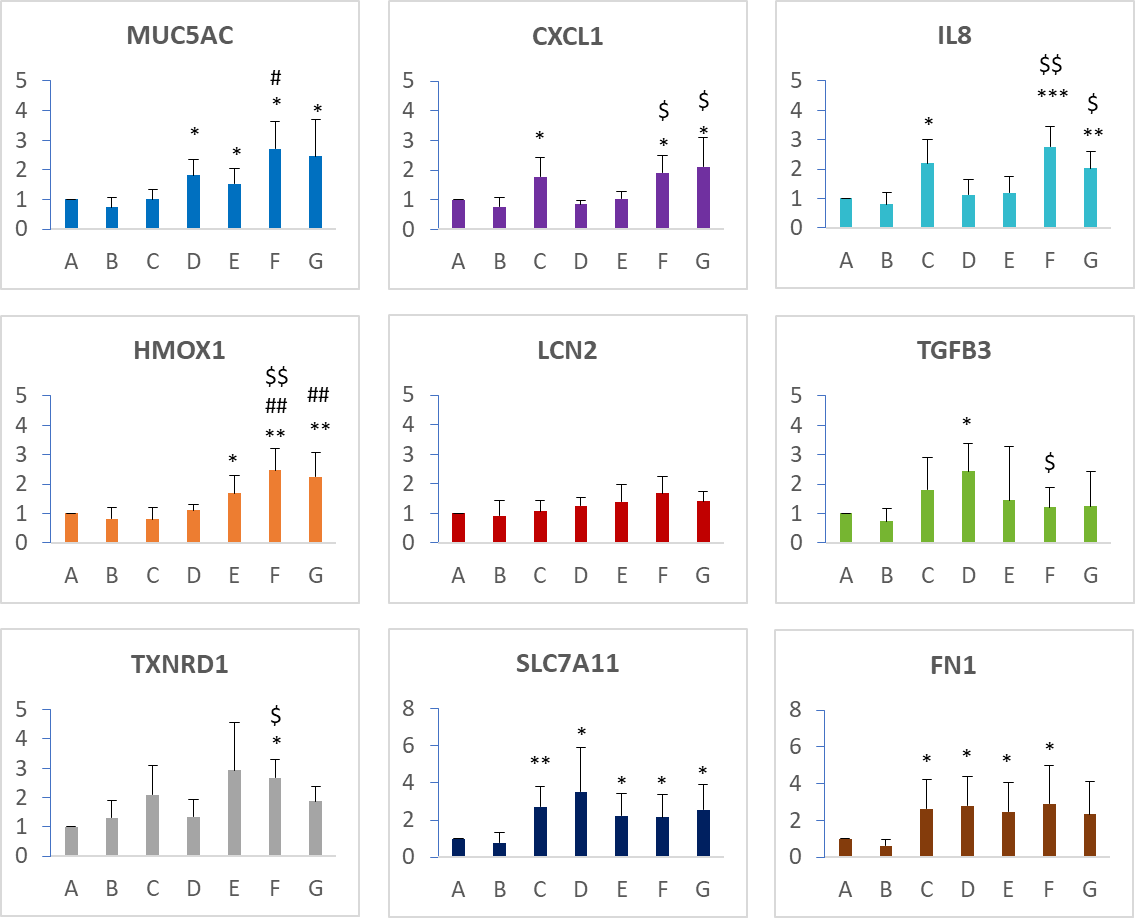


**Supplementary Figure S13** Expression of selected genes in SmallAir at 1 day following various exposures (A: Incubator control; B: System control – dried H_2_O aerosol exposure; C: Bleomycin + dried H_2_O aerosol exposure; D: CeO_2_NP_LD (low - 275 ± 77 ng/cm^2^); E: CeO_2_NP_HD (high - 706 ± 151 ng/cm^2^); F: Bleomycin + CeO_2_NP_LD; G: Bleomycin + CeO_2­_NP_HD. Gene expression alterations were normalized to those of the respective controls from individual donors. The data are shown as the means ± SDs. Statistical significance was assessed via paired t tests. Compared with condition B (system control, dried H_2_O aerosol exposure), the difference was statistically significant (* p < 0.05, ** p < 0.01, *** p < 0.001). Compare with condition C (bleomycin + dried H_2_O aerosol exposure), the difference was statistically significant (# p < 0.05, ## p < 0.01). Compared with condition D/E (respective CeO_2_NP aerosol exposures), the difference was statistically significant ($ p < 0.05, $$ p < 0.01).

**References**

1. Chen, H.-S., et al. *Synthesis and characterization of nano ceria for biological applications*. in *Nano-Bio Sensing, Imaging and Spectroscopy*. 2013. SPIE.

2. Schneider, C.A., W.S. Rasband, and K.W. Eliceiri, *NIH Image to ImageJ: 25 years of image analysis.* Nat Methods, 2012. **9**(7): p. 671-5.

3. Guo, C., et al., *Pulmonary toxicity of inhaled nano-sized cerium oxide aerosols in Sprague-Dawley rats.* Nanotoxicology, 2019. **13**(6): p. 733-750.

4. Buckley, A., et al., *Slow lung clearance and limited translocation of four sizes of inhaled iridium nanoparticles.* Particle and Fibre Toxicology, 2017. **14**(1): p. 5.

5. Whalen, J.E., Foureman, G. L., and Vandenberg, J. J., *Inhalation Risks Assessment at the Environmental Protection Agency*, in *Inhalation Toxicology* e. H. Salem and S. A. Katz, Taylor and Francis, Editor. 2006, CRC Press. p. 3–38.

6. Mauderly, J.L., *Respiration of F344 rats in nose-only inhalation exposure tubes.* J Appl Toxicol, 1986. **6**(1): p. 25-30.

7. Filho, W.J., R.G. Fontinele, and R.R. de Souza, *Reference database of lung volumes and capacities in wistar rats from 2 to 24 months.* Curr Aging Sci, 2014. **7**(3): p. 220-8.

8. Semmler-Behnke, M., et al., *Nanoparticle delivery in infant lungs.* Proc Natl Acad Sci U S A, 2012. **109**(13): p. 5092-7.

9. Asgharian, B., Price, O., Miller, F., Subramaniam, R., Cassee, F. R., Freijer, J., van Bree, L., de Winter-Sorkina, R. , *MPPD, Multiple-Path Dosimetry Model v2.11. Applied Research Associates (ARA), The Hamner Institutes for Health Sciences, the National Institute of Public Health and the Environment (RIVM), the Netherlands, and the Ministry of Housing, Spatial Planning and the Environment, the Netherlands.* 2009.

10. Buckley, A., et al., *Aerosol exposure at air-liquid-interface (AE-ALI) in vitro toxicity system characterisation: Particle deposition and the importance of air control responses.* Toxicology in Vitro, 2024. **100**: p. 105889.

11. Paton, C., et al., *Iolite: Freeware for the Visualisation and Processing of Mass Spectrometric Data.* J. Anal. At. Spectrom. VL - IS -, 2011. **online**.
